# Mutations in CFAP57 disrupt the localization of MYH10 and IFT88, leading to flagellogenesis failure in humans and mice

**DOI:** 10.1186/s40246-025-00859-x

**Published:** 2025-12-29

**Authors:** Yongjie Chen, Lin Li, Ranran Meng, Shanze Li, Yuhua Li, Zhaodi Jiang, Dan Xu, Zhao Lu, Chenghong Yin, Yanwei Sha, Fengchao Wang

**Affiliations:** 1 Central Laboratory, Beijing Obstetrics and Gynecology Hospital, Capital Medical University, Beijing Maternal and Child Health Care Hospital, Beijing, 100026 China; 2 National Institute of Biological Sciences, Beijing, 102206 China; 3 Tsinghua Institute of Multidisciplinary Biomedical Research, Tsinghua University, Beijing, 102206 China; 4 Capital Medical University, Beijing, China; 5 Department of Reproductive Medicine, Department of Obstetrics and Gynecology, Women and Children’s Hospital, School of Medicine, Xiamen University, Xiamen, China; 6 Xiamen Key Laboratory of Reproduction and Genetics, Xiamen, China; 7 Fujian Provincial Key Laboratory of Reproductive Health Research, Xiamen University, Xiamen, Fujian China

**Keywords:** CFAP57, MYH10, IFT88, MMAF, Male infertility, ICSI

## Abstract

Flagellogenesis in mammalian sperm is essential for sperm motility and successful egg fertilization. Multiple morphological abnormalities of the sperm flagella (MMAF) represent a condition characterized by various structural defects in the flagellum. While CFAP57 has been identified as a factor in the pathogenic mechanisms of MMAF, the precise molecular regulation underlying this process remains unclear. Here, we report novel biallelic mutations in the CFAP57 gene identified in two infertile males from two unrelated families. The first patient carried a homozygous nonsense mutation in *CFAP57* [NM_001195831.2: c.3250 C > T (p.R1084X)], while the second was compound heterozygous for two missense mutations c.1340T > C (p.V447A) and c.1856G > A (p.R619H). CRISPR-Cas9-generated CFAP57 mutant mice recapitulated human MMAF phenotypes, exhibiting structural flagellar defects and complete infertility. Using immunoprecipitation-mass spectrometry (IP-MS), we identified MYH10, a non-muscle myosin II isoform, as an interaction partner of CFAP57. Immunofluorescence analysis confirmed that both MYH10 and CFAP57 localize to the sperm flagella. We further examined the precise localization of MYH10 at the ultrastructural level using immunoelectron microscopy. Gold particles conjugated to the MYH10 antibody were predominantly detected in the sperm flagella. In sperm with CFAP57 mutations, MYH10 was mislocalized to the mid-piece region while being notably absent from the principal and end pieces. This mislocalization affected the expression of IFT88, a key component of the intraflagellar transport (IFT) system that plays a critical role in mammalian flagellar assembly. Fortunately, ICSI can overcome CFAP57-associated male infertility. Together, our findings establish CFAP57 as an important mediator of sperm flagellogenesis that orchestrates MYH10 and IFT88 positioning and intraflagellar transport dynamics to maintain flagellar integrity, providing molecular insights into MMAF-associated male infertility.

**Supplementary Information:**

The online version contains supplementary material available at 10.1186/s40246-025-00859-x.

## Introduction

Infertility, a multifactorial pathological condition, impacts approximately 10%–15% of couples worldwide, with male infertility contributing to nearly 50% of all cases [1, 2]. During the differentiation of haploid germ cells into mature spermatozoa, a process known as spermiogenesis, a specific set of molecules involved in chromatin remodeling, acrosome biogenesis, and flagellar morphogenesis is expressed in a tightly regulated, stage-dependent manner. The morphogenesis and assembly of the flagellar apparatus in mammalian spermatozoa constitutes a fundamental developmental process that is indispensable for establishing progressive motility and consequent fertilization capacity. The sperm flagellum exhibits a distinct quadripartite architecture that extends from the connecting piece adjacent to the sperm head through the mitochondria-encased midpiece and fibrous sheath-reinforced principal piece to the terminal end piece. At its core lies the axoneme, which contains the highly conserved “9 + 2” microtubule arrangement—nine peripheral doublets surrounding a central pair. Cross-sectional analysis reveals three key structural components: radial spokes (RS), which connect to and regulate the central pair (CP), inner dynein arms (IDAs) that coordinate microtubule sliding, and outer dynein arms (ODAs) that provide the driving force for motility. Defective flagellar development compromises sperm quality, resulting in multiple morphological abnormalities of the sperm flagella (MMAF). This disorder encompasses various flagellar defects, including complete absence, shortening, coiling, bending, and irregular shapes [1, 3].

Emerging genomic evidence has elucidated pathogenic variants contributing to MMAF-related infertility, including dynein axonemal heavy chain (DNAH) family members (e.g., DNAH1, DNAH2, DNAH6, DNAH7, DNAH8, DNAH10 DNAH12 and DNAH17) [3–9]; Cilia and flagella associated protein (CFAP) family members (e.g., CFAP43, CFAP44, CFAP47, CFAP57, CFAP58, CFAP61, CFAP65, CFAP69, CFAP70, CFAP91, CFAP135 and CFAP251) [6, 9–17]; Coiled-coil domain-containing (CCDC) protein family members (e.g., CCDC34, CCDC39, CCDC42, CCDC63, CCDC113, CCDC172 and CCDC189) [9, 18–22]. Additionally, mutations in ZMYND12, FSIP2, AKAP3/4, ARMC2, QRICH2, SPEF2, TTC21A, TTC29, TMEM232 and CABCOCO1 also lead to MMAF [9, 18, 21, 23–29]. Although MMAF represents a relatively common autosomal recessive disorder, the underlying genetic causes remain unknown in a significant proportion of cases [18].

The assembly and function of the sperm flagellum require the precise orchestration of various structural and regulatory proteins. Several key molecules have been implicated in ensuring flagellar integrity and motility through their roles in axonemal architecture, microtubule stability, and protein transport. For example, DNAH1 was the first identified MMAF gene, DNAH1 mutation impaired the inner dynein arm assembly [3]. CFAP58 localizes along the entire flagellum and its deficiency leads to reduced levels of the axonemal markers SPAG6 and SPEF2, as well as HSP60, a mitochondrial sheath component, in spermatozoa from individuals harboring CFAP58 variants [12]. Additionally, CCDC189 and CABCOCO1 interact with the radial-spoke-specific protein RSPH1, and notably associate with intraflagellar transport (IFT) components IFT20 and IFT88, underscoring the potential involvement of the IFT machinery in radial spoke assembly and flagellogenesis [18]. Similarly, FSIP2 disruption not only abolishes AKAP4 expression but also causes mislocalization or depletion of multiple IFT-B complex members, including IFT88, IFT74, and IFT20, suggesting that defects in IFT-related pathways contribute to the MMAF phenotype [26, 30]. Furthermore, proteins such as TMEM232 and QRICH2 regulate flagellar microtubule stability and protein expression, respectively, further emphasizing the complexity of molecular networks governing flagellogenesis [25, 28]. Additionally, CEP112 has been shown to localize at the neck region and atypical centrioles of mature sperm, with Cep112-knockout mice recapitulating key features of human asthenoteratozoospermia. Mechanistically, CEP112 forms RNA granules during spermiogenesis and undergoes liquid-liquid phase separation (LLPS) to assemble biomolecular condensates that selectively enrich target mRNAs essential for sperm development, including *Fsip2*, *Cfap61*, and *Cfap74*—genes directly implicated in flagellar assembly and IFT-related processes. These findings highlight the multi-layered regulatory networks—from protein transport to RNA metabolism—that govern the formation and function of the sperm flagellum [31].

Human CFAP57 encodes four transcript isoforms according to the NCBI database (https://www.ncbi.nlm.nih.gov/gene/149465): NM_001167965.1 (1-1239aa), NM_001195831.3 (1-1250aa), NM_001378189.1 (1-1250aa), and NM_152498.3 (1-698aa). Previous studies have demonstrated that the long transcript isoform (NM_001195831.3) is essential for sperm flagellar assembly, whereas the short transcript isoform (NM_152498.3) plays a critical role in respiratory ciliary function [11, 32]. Although mutations in the CFAP57 gene have been implicated in MMAF, the molecular mechanisms underlying CFAP57-mediated flagellar assembly remain poorly understood. In this study, we identified novel CFAP57 gene mutations in two unrelated infertile males: the first patient (P1) carrying a homozygous nonsense mutation [NM_001378189.1: exon 20: c.3250 C >T (p.R1084X), NM_001195831.3:exon 21:c. 3349 C >T (p.R1117X)] and the second patient (P2) with compound heterozygous missense mutations [NM_001167965:exon 8: c.1340T >C (p.V447A), NM_001195831:exon 8: c.1340T >C (p.V447A), NM_152498:exon 8: c.1340T >C (p.V447A) and NM_001167965:exon 11: c.1856G >A (p.R619H), NM_001195831:exon 11: c.1856G >A (p.R619H), NM_152498:exon 11: c.1856G >A (p.R619H)]. We propose that the P1 mutation may selectively affect the long CFAP57 transcript isoform (NM_001378189.1 and NM_001195831.3), whereas the P2 mutation may disrupt both the long and short transcript isoforms (NM_001195831.3 and NM_152498.3), ultimately resulting in abnormal spermatogenesis. Indeed, spermatozoa from these individuals, as well as from CFAP57^R1083X^ mutant mice (*Cfap57*^*M/M*^), displayed coiled, curved, and shortened flagella. Mechanistically, we demonstrated that CFAP57 physically interacts with MYH10 and is indispensable for its proper localization within the sperm tail. Loss of CFAP57 leads to the mislocalization of MYH10 and IFT88 at the mitochondrial sheath and flagellum, thereby disrupting intraflagellar transport and impairing flagellar assembly. Collectively, these findings not only clarify the role of CFAP57 in MMAF pathogenesis but also reveal a previously unrecognized function of MYH10 in sperm flagellogenesis, providing new insights into the molecular etiology of male infertility and highlighting potential therapeutic targets.

## Materials and methods

### Patients

Two infertile Han Chinese men with fertilization failure were recruited from the Department of Andrology, Women and Children’s Hospital, School of Medicine, Xiamen University (Xiamen, Fujian, China). The patients have low sperm motility, high sperm malformation rate, abnormal tail morphology, and abnormal sperm DNA fragmentation etc. Based on comprehensive evaluation of all indicators, the patients were diagnosed with MMAF syndrome. Routine analysis was performed following the guidelines of the World Health Organization Laboratory Manual for the Examination and Processing of Human Semen (5th edition). Physical examination of patient showed normal development of the male external genitalia, normal bilateral testis size, and no abnormality in the bilateral spermatic veins upon palpation. This study was approved by the Ethics Committees of the Women and Children’s Hospital, School of Medicine, Xiamen University (KY-2023-098-H01). The patients recruited for this study signed a written informed consent form.

### WES and in-silico bioinformatic analysis

Whole-exome sequencing (WES) of the genomic DNA extracted from patients with asthenozoospermia was carried out as described previously [33]. Briefly, Genomic DNA was extracted from whole peripheral blood samples from each patient using the QIAamp DNA Blood Kit (Qiagen, Valencia, CA, USA), according to the manufacturer’s protocol. Sanger sequencing was performed to validate the sequence variants in each patient. Primers used for PCR and sequencing are listed in Table S1.

### Animals

Mice were housed in the National Institute of Biological Sciences, according to the Ministry of Health National guidelines for Housing and Care of Laboratory Animals. The animal use protocol was approved by the Institutional Animal Care and Use Committee of National Institute of Biological Sciences. C57BL/6 wild-type (WT) mice were obtained from Beijing Vital River Laboratory Animal Technology Co. Ltd. The guide RNA (gRNA) targeting CFAP57^R1083^, which sequence was ‘ctttgagaagtacgtgcagcggg’. The gRNA was transcribed using the T7 promoter in vitro. The donor sequence was ‘gaacccgggctgctgaaggagaagatccgtggcctctttgagaagtacgtgcagTAAgcagacatggtgagctctgagcgccccgccctc’. *Cfap57*^*M/M*^ mice were generated by co-microinjection of Cas9 protein, gRNA and donor sequence into C57BL/6 zygotes. Chimeras were then crossed with WT female C57BL/6 mice. Germline transmission was confirmed by genotyping PCR and sanger sequencing. The primers listed in Supplemental Table S2.

### Fertility assessment and testis histology

For fertility assessment, WT or *Cfap57*^*M/M*^ males were co-housed with WT females at a 2:1 ratio (*n* = 6 males per genotype) for 3 months. Daily monitoring for post-coital vaginal plugs was performed to confirm mating success, with plug formation frequency documented throughout the study period. Upon detection of vaginal plugs, pregnant females were immediately separated from males, and the resulting offspring were analyzed for number and viability. Testis from male adults (*Cfap57*^*M/M*^ and WT) were fixed in Davidson’s fixative solution for histological analysis. These samples were sectioned, rehydrated, and stained with hematoxylin and eosin.

### Human and mice semen parameter analysis

Routine semen analysis included evaluation of semen volume, sperm concentration, total motility, and percentage of morphologically normal spermatozoa. Mouse sperm was obtained from the caudal epididymis of WT (10–12 weeks), but not the *Cfap57*^*M/M*^ males. Each WT caudal epididymis was placed in 300 µL of human tubal fluid (HTF) medium for capacitation for 30 min at 37 °C, *Cfap57*^*M/M*^ caudal epididymis was Cut into pieces in HTF medium, then collected the supernatant and centrifuge at 3000 rpm for 5 min. Sperm morphology was analyzed by firstly fixing in 2% paraformaldehyde (PFA). Hematoxylin and eosin (H&E) staining was carried out to assess the morphology of spermatozoa.

For Patient 2 (P2), we were only able to obtain genetic sequencing results and clinical phenotypic data, as the sperm samples were unfortunately lost during storage and the patient became unreachable for follow-up sampling. The functional studies were performed using P1 samples exclusively due to P2 sample unavailability.

### Western blotting

Human semen samples and mouse testis were lysed in RIPA buffer [50 mM Tris-HCl (pH 7.4), 150 mM NaCl, 1% NP-40, 1 mM EDTA, and 0.1% sodium dodecyl sulfate] containing a protease inhibitor cocktail (4693124001; Roche). Each sample was kept on ice for 30 min and centrifuged at 14,000 rpm for 20 min at 4 °C. Quick Start Bradford protein 1× dye (#500 − 0205; Bio-Rad) was used to determine the protein concentration. A total of 20 µg of protein was resolved by electrophoresis on 4–20% precast gels and transferred to a polyvinylidene difluoride membrane (Millipore, Darmstadt, Germany). Membranes were blocked with 5% skim milk in Tris-buffered saline containing 0.1% Tween 20 for 1 h and then incubated overnight with primary antibodies diluted in antibody diluent (WB500D; NCM) at 4 °C. Antibodies specific for CFAP57 (#HPA028623,1:1000 dilution; it specifically binds to amino acids 1165–1250 of human CFAP57, sigma-aldrich, USA), α-Tubulin (#AC012, 1:2000; ABclonal Technology, China), and GAPDH (#AC033, 1:10000; ABclonal Technology, China) served as loading controls. The samples were then incubated with the appropriate secondary antibodies conjugated to HRP (#AS014 or AS003, 1:10000; ABclonal Technology, China) for 1 h. The protein bands were detected using an ECL detection reagent.

### Isolation of testicular spermatids

Isolation of different types of spermatids in testis, were performed under standard protocols as previously described [33, 34]. In brief, WT and *Cfap57*^*M/M*^ testis were collected from 2 months-old mice, and sequential digestions with collagenase IV and trypsin. Next, different germ cells types were isolated through the discontinuous bovine serum albumin (BSA) density gradient. The pachytene spermatocyte, round spermatid, and elongating spermatid were collected and fixed in 4% paraformaldehyde (PFA) for Immunofluorescence analysis.

### Immunofluorescence staining

Sperm were spread on glass slides for morphological observation or immunostaining. For immunofluorescence, sperm smears were stained with primary antibodies specific to CFAP57 (#HPA028623,1:200 dilution; sigma-aldrich, USA), IFT88 (#13967-1-AP, 1:200, Proteintech, China), MYH10 (#19673-1-AP, 1:200, Proteintech, China), α-Tubulin (#AC012, 1:500; ABclonal Technology, China). The sections were stained with Alexa Fluor 488, 555 and 647- conjugated IgG (#A-21202, #A-31572, and #A21245, 1:1000; Invitrogen, USA) and peptide nucleic acid (PNA; #L738, 1:1000; Sigma-Aldrich, USA) or before being counterstained with DAPI (#D1306, 1:1000; Invitrogen, USA). Immunofluorescence images were captured using an A1 laser scanning confocal microscope (N-SIM E; Nikon).

### Scanning electron microscopy (SEM) and transmission electron microscopy (TEM)

Human and mice sperm were prepared for SEM and TEM as described previously [35]. Briefly, for the SEM assay, spermatozoa were fixed in 4% PFA overnight at 4 °C. Specimens were then deposited on poly L-lysine-coated coverslips and air-dried. Subsequently, the coverslips were dehydrated via an ascending gradient of ethanol concentrations at room temperature, coated with gold particles, and observed with a Zeiss EVO LS10 SEM.

For TEM, samples including testis, seminiferous tubules, and sperm cells were fixed with 2.5% glutaraldehyde (G5882, Sigma-Aldrich, USA), which was followed by treatment with 1% OSO4 for 1 h and with 2% uranyl acetate overnight at 4 °C. Subsequently, dehydration was performed using a series of graded acetone solutions by the progressive lowering temperature method. Then, the samples were infiltrated and embedded in Epon 812. Ultrathin (90 nm in thickness) sections were stained with 3% uranyl acetate and lead citrate prior to TEM using a Philips FEI Tecnai G2 Spirit instrument with an acceleration voltage of 120 kV.

### Immunoelectron microscopy (IEM)

For Immunoelectron microscopy (IEM), mouse sperms were fixed in 4% PFA for 2 h before cryo-immobilizing by HPF ((HPF COMPACT 01). After freeze-substitution in acetate containing 0.1% Uranium acetate, the sperms were infiltrated and embedded in LRW according to the instructions. 90 nm thick slices were mounted on nickel grids for immunolabeling. The slices were incubated with primary antibodies MYH10 (#19673-1-AP, 1:50, Proteintech) and IgG (#AC005, 1:50; ABclonal Technology, China), followed by gold-conjugated secondary antibodies. The sections were placed in nickel mesh for TEM.

### Immunoprecipitation and mass spectrometry

Testis from WT and *Cfap57*^*M/M*^ were suspended in lysis buffer containing 50 mM Tris-HCl (pH 7.4), 150 mM NaCl, 1% NP-40, 1 mM EDTA, 0.1% sodium dodecyl sulfate and protease inhibitor cocktail (4693124001; Roche, Germany) for 30 min on ice. After centrifugation at 14,000 rpm, at 4 °C for 30 min, the supernatant was incubated with CFAP57 (#HPA028623,1:100 dilution; sigma-aldrich, USA) at 4 °C overnight and subsequently incubated with protein G agarose beads (#P2105-5 ml, Beyotime, China) for 4 h at 4 °C with gentle rotation. After five times of washing with the same lysis buffer, the protein samples were denatured with SDS-PAGE Sample Loading Buffer (5x) (#RM00001, ABclonal Technology, China) at 100 °C for 10 min.

The samples were separated on SDS-PAGE followed by silver staining (Sigma, PROTSIL1). The stained proteins were destained and in-gel digested with trypsin (10 ng mL-1 trypsin, 50 mM ammonium bicarbonate, pH 8.0) overnight at 37℃. Peptides were extracted with 5% formic acid/50% acetonitrile and 0.1% formic acid/75% acetonitrile sequentially and then concentrated to 20 ul. The extracted peptides were separated by an analytical capillary column (100 μm *15 cm) and packed with 3 μm spherical C18 reversed phase material (YMC, Kyoyo, Japan). A Waters nano Acquity UPLC system was used to generate the following HPLC gradient: 0%–30% B in 60 min, 30%–70% B in 15 min (A = 0.1% formic acid in water, B = 0.1% formic acid in acetonitrile). The eluted peptides were sprayed into an LTQ ORBITRAP Velos mass spectrometer (Thermo Fisher Scientific, San Jose, CA, USA) equipped with a nano-ESI ion source. The mass spectrometer was operated in data-dependent mode with one MS scan followed by ten HCD (High-energy Collisional Dissociation) MS/MS scans for each cycle. Identified peptides were searched in the IPI (International Protein Index) Mouse protein database on the Mascot server (Matrix Science Ltd, UK). The search parameters are 10 ppm mass tolerance for precursor ions and 0.02 Da mass tolerance for product ions; two missed cleavage sites were allowed for trypsin digestion. Methionine oxidation was set as variable modification. The search results were filtered with both peptide significance threshold and expectation value (< 0.05). The MS analysis was performed at the Proteomics Center of the National Institute of Biological Sciences in Beijing.

To validate the mass spectrometry data, MYH10 and RAB1, along with rabbit IgG antibodies, were incubated with the WT testis lysate supernatant at 4 °C overnight. Afterward, protein G agarose beads (#P2105-5 ml, Beyotime, China) were added and incubated for an additional 4 h. The magnetic beads were washed 5 times with the same lysis buffer, followed by the addition of 80 µL of lysis buffer and 20 µL of 5 x loading buffer, which were boiled at 100 °C for 10 min and then analyzed using SDS-PAGE. Antibodies MYH10, RAB1, and CFAP57 were incubated separately for Western blotting detection.

### Protein structural and domain analysis

Three-dimensional protein structure prediction was conducted using AlphaFold2 (https://alphafold.ebi.ac.uk/, UniProt ID: Q96MR6). Mutation sites were mapped onto the predicted structure using PyMOL.

Protein domain predictions were performed using multiple complementary approaches: (i) UniProt database annotations (https://www.uniprot.org/uniprotkb/Q96MR6), (ii) InterPro domain analysis (https://www.ebi.ac.uk/interpro/), and (iii) SMART database predictions (http://smart.embl-heidelberg.de/), and visualized using the online tool (https://prosite.expasy.org/mydomains/). The final vector graphic was then recreated at a 1:1 scale using Adobe Illustrator 2020.

### Sperm protein fractionation extraction

Sperm cells collected from the cauda epididymis were subjected to sequential protein extraction following a modified protocol [36–38]. First, sperm were resuspended in 1% Triton X-100 buffer (50 mM NaCl, 20 mM Tris-HCl pH 7.5, protease inhibitors) and incubated at 4 °C for 60 min. After centrifugation at 13,000 rpm for 10 min, the supernatant (Triton-soluble fraction) was collected. Second, the pellet was extracted with 1% SDS buffer (75 mM NaCl, 24 mM EDTA pH 6.0) at room temperature for 60 min, followed by centrifugation to obtain the SDS-soluble fraction (supernatant). Finally, the remaining pellet was boiled in 2% SDS buffer (66 mM Tris-HCl, 10% glycerol, 0.005% bromophenol blue) for 5 min and centrifuged to yield the SDS-resistant fraction.

### Statistical analyses

Statistical analyses were performed using GraphPad Prism 8. Data are expressed as the mean ± SEM. Statistical analyses of the difference between two groups were performed using standard Student’s t test. p-values < 0.05 (denoted by * in Figures) and < 0.01 (denoted by ** in Figures) were considered statistically significant.

## Results

### Identification of the mutations in CFAP57 among men with MMAF

**Fig. 1:**
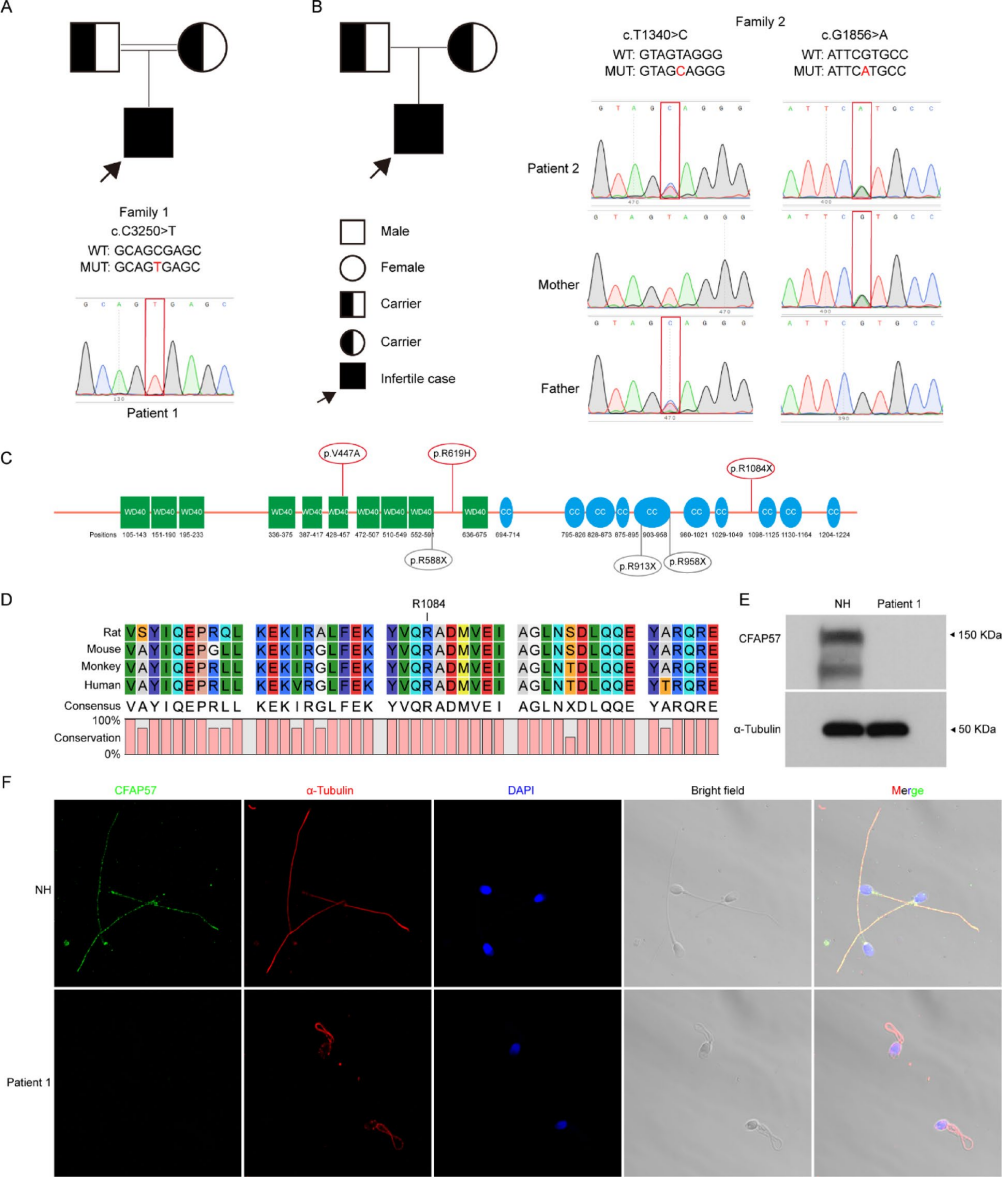
Two novel variants of *CFAP57* in the patients with MMAF. **A** and **B** Pedigrees of the two families affected by the variants in *CFAP57*. The black squares represent the affected individuals. The red rectangles show mutated locations in the validation of Sanger sequencing. **C** Domain architecture showing mutated positions within CFAP57 proteins. Red ovals represent the mutated positions identified in this study, while gray ovals represent previously reported mutated positions. **D** The mutated position of *CFAP57* from P1 [c.3250 C > T (p.R1084X)] is conserved among species. **E** Western blotting confirmed the loss function of CFAP57 protein. with α-Tubulin as a loading control. **F** Immunofluorescence analysis of CFAP57 (green) and α-Tubulin (rad) expression in spermatozoa flagella. Western blotting and immunofluorescence data shown are from P1 samples only. Scale bar: 5 μm. WT, wild type; MUT, CFAP57 mutations; NH, normal human; MMAF: multiple morphological abnormalities of the sperm flagella

Here, two infertile cases were recruited for the project. With the patient’s consent, Whole-exome sequencing (WES) was performed on the infertile patient. We identified the first patient (P1) with a homozygous stop-gain mutation in *CFAP57*, c.3250 C > T (p.R1084X), born from a consanguineous family (Fig. 1A). A second patient (P2) harbored a rare compound heterozygous mutation in *CFAP57*, with c.1340T > C (p.V447A) inherited from the father and c.1856G > A (p.R619H) from the mother (Fig. 1B). Sanger sequencing validated all variants in the infertile patients, suggesting an autosomal recessive mode of inheritance (Fig. 1A and B).

The CFAP57 protein contains multiple WD40 repeat structures and coiled-coil domains (Fig. 1C). A pathogenic CFAP57 mutation [c.1762 C >T (p.R588X)] was previously reported in a PCD patient with unknown fertility status [32]. A newly report has shown that two truncation mutations at the coiled-coil region [c.2872 C >T (p. R958X) and c.2737 C >T (p. R913X)], were associated with male infertility [11]. Our domain mapping analysis revealed distinct mutation patterns between patients (Fig. 1C). The first patient’s nonsense mutation [c.3250 C >T (p.R1084X)] is positioned in an interdomain region, located near the coiled-coil domains, and exclusively affects the long transcript isoform (Fig. 1C, Supplementary Fig. 1A). In contrast, mutations in the second patient showed differential domain localization: c.1340T >C (p.V447A) maps within the WD40 repeat domain, while c.1856G >A (p.R619H) is situated in the interdomain linker region between WD40 repeats (Fig. 1C, Supplementary Fig. 1B). We speculated that mutations at V447 and R619 could disrupt both the conserved WD40 domain and C-terminal coiled-coil domains, while R1084 might eliminate critical C-terminal coiled-coil domains. These findings suggest that multiple mutation sites within the CFAP57 may contribute to male infertility.

CFAP57 is highly conserved across ciliated species, consistent with its fundamental role in motile ciliary function, as previously demonstrated by Bustamante-Marin et al. [32]. The human CFAP57 shares 98.40%, 87.67%, and 87.99% of protein identity with its orthologs in rhesus monkeys, rats and mice (Supplementary Fig. 1C), respectively (https://www.uniprot.org/). The amino acid positions of the three CFAP57 missense variants were highly conserved across humans, monkeys, rats, and mice (Fig. 1D, Supplementary Fig. 1D).

The semen characteristics from P1 and P2 was analyzed by routine clinical tests, and the results revealed that the sperm motility was significantly reduced, and most sperm were classified as immobile sperm (64.1%-79.3%) (Table 1). Sperm morphology showed that most spermatozoa had malformed flagella, including absent, short, coiled, bent, and irregular caliber, which are typical characteristics of MMAF (Table 1). DNA fragmentation index analysis revealed significant sperm DNA fragmentation, indicating compromised chromatin integrity in patients with CFAP57 mutations. We further found that these mutations in CFAP57 reduced its expression in patient sperm (Fig. 1E). Immunofluorescence staining showed that CFAP57 and α-tubulin co-localized in the sperm flagellum, but it was disappeared in patient sperm flagellum (Fig. 1E). These findings indicate that the novel CFAP57 mutations may influence spermiogenesis.

**Table 1:** Semen characteristics and sperm motility in men with CFAP57 mutation

Characteristic	P1	P2	Reference
Semen parameters			
Volume (ml)	2.5-3.0	2.2–4.2	≥ 1.5 ml
Concentration (10^6^/ml)	29.8–44.1	26.9–35.9	≥ 15 × 10^6^/ml
Progressive motility (%)	14–19.7	15.9–21.8	≥ 32%
Non-progressive motility (%)	3.9–6.7	11.0-14.1	–
Immobile sperm (%)	76.4–79.3	64.1–73.1	–
Sperm morphology			
Normal flagella (%)	6.5	5.8	> 23.0
Absent flagella (%)	10.2	9.6	< 5.0
Short flagella (%)	48.5	45.6	< 1.0
Coiled flagella (%)	12	13	< 17.0
Bent (%)	10.6	13.5	< 13.0
Irregular caliber (%)	12.2	12.5	< 2.0
Sperm DNA parameters			
DNA Fragmentation Index	32.93	28.76	< 15%
High DNA Stainability	10.31	12.56	< 15%

Lower and upper reference limits are shown according to the World Health Organization standards and the distribution ranges of morphologically abnormal spermatozoa observed in fertile individuals

**Fig. 2:**
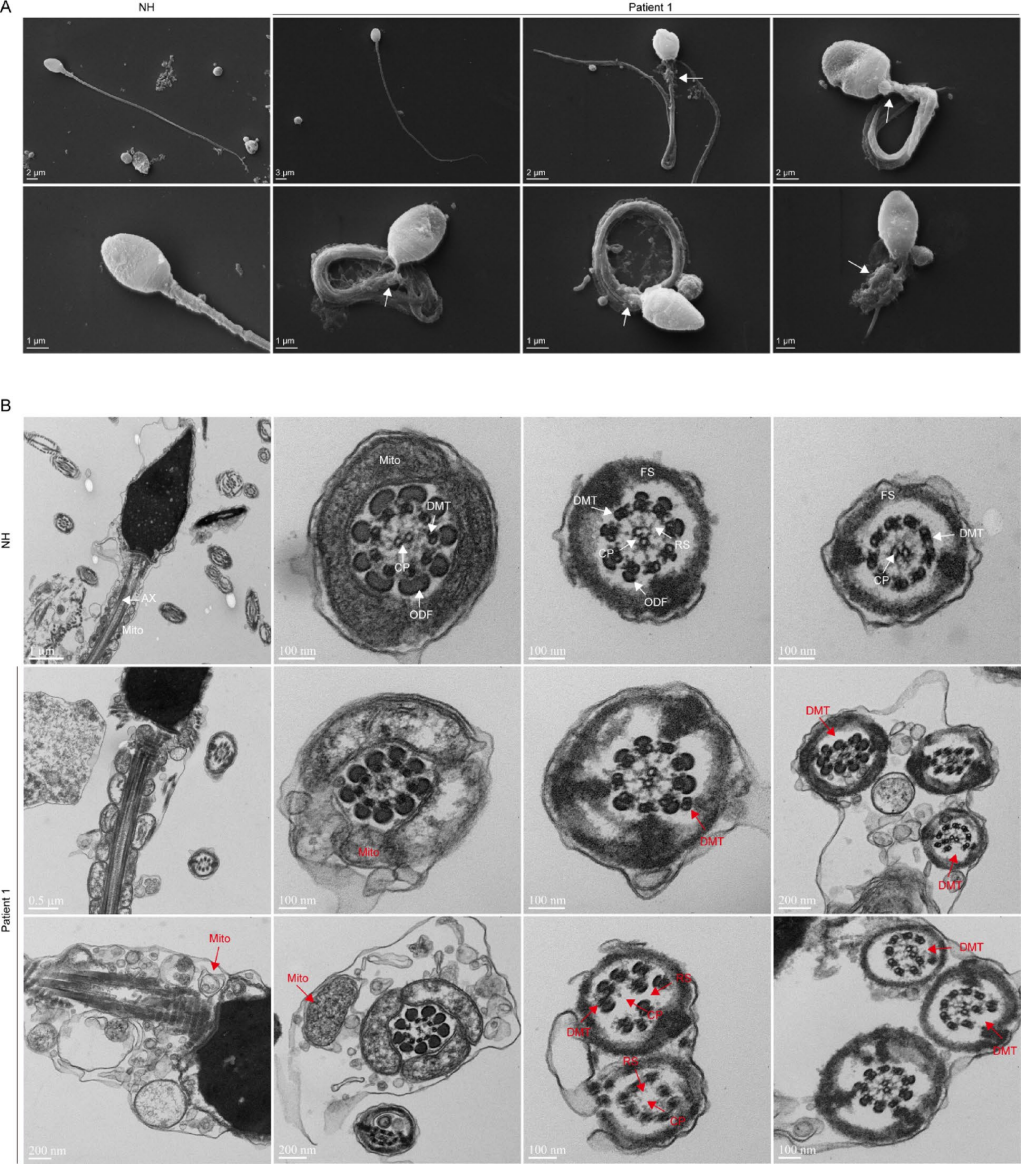
Morphology and ultrastructure of the spermatozoa from patient 1 affected by CFAP57 mutations. **A** SEM showed morphologically characteristics of P1’ spermatozoa flagella defect. The white arrow indicates the abnormal mitochondria and axoneme. **B** TEM images of spermatozoa cross-sections revealed mitochondrial disorganization, absence of the central pair (CP) and radial spokes (RS), and displacement of the doublet microtubules, as indicated by red arrowheads. Abbreviations: Ax, axoneme, Mito, mitochondria; ODF, outer dense fibers; DMT, doublet microtubule; CP, central pair; RS, radial spokes

To gain a more detailed understanding of the ultrastructural morphology of sperm in patient 1 with CFAP57 mutation, we first examined the morphological structure of sperm from both normal and P1 using scanning electron microscopy (SEM). Morphological analysis demonstrated that sperm heads in the normal group exhibited a characteristic oval morphology, containing a densely packed nuclear structure. The midpiece, which connects to the sperm head, contained densely packed and well-organized mitochondria, while the tail exhibited considerable length and a smooth outer membrane surface. In contrast, the sperm from P1 exhibited various morphological abnormalities, including irregular shapes such as indentation and conical forms in the heads. The midpiece showed pronounced mitochondrial aggregation and disorganized arrangement. The tail exhibited characteristic MMAF phenotypes, including coiled, bent, irregular, short or/and absent flagella (Fig. 2A). Furthermore, we examined the flagella structure by using transmission electron microscopy (TEM). In contrast to the regular positioning of the mitochondrial and “9 + 2” microtubules, the sperm with the CFAP57 mutation displayed disorganization in mitochondrial assembly and haphazard “9 + 2” microtubules arrangement, for example, the CP and RS are absent, the doublet microtubule (DMT) was displacement (Fig. 2B). It should be noted that a minority of sperm maintained relatively intact flagella morphology by SEM and TEM analysis. These results indicate CFAP57 is essential for the development of sperm flagellum.

### Male mice carrying CFAP57 stop-gain mutations are infertile

**Fig. 3:**
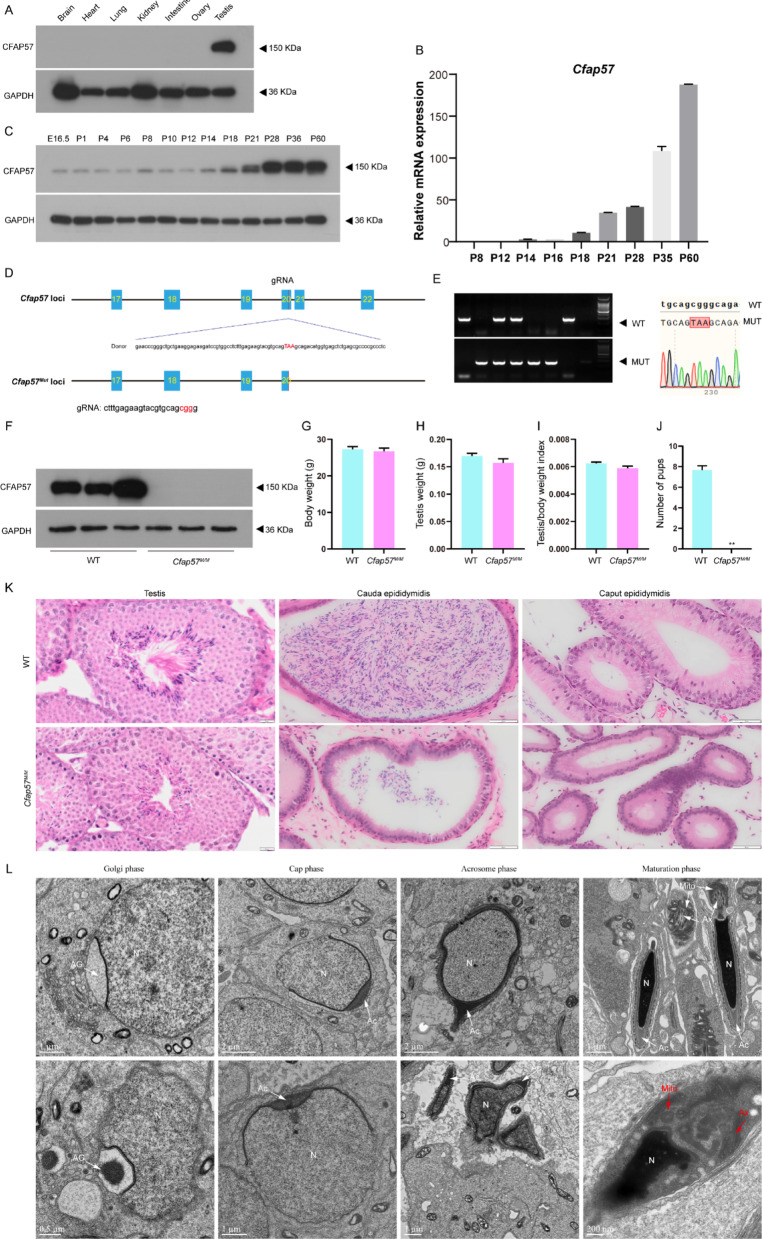
Phenotype analysis of *Cfap57*^*M/M*^ male mice. **A** Western blotting showed CFAP57 was highly expressed in mouse testis. GAPDH as a loading control, (*n* = 3), Western blot analysis using anti-CFAP57 antibody (HPA028623, epitope aa 1165–1250). **B** qPCR analysis of *Cfap57* mRNA expression in testes at different developmental stages, with 18 S rRNA serving as a loading control, *n* = 3, Data were presented as the mean ± SEM. **C** Western blotting analysis of CFAP57 expression in E16.6 and postnatal mouse testes. GAPDH as a loading control, *n* = 3–6. **D** Schematic diagram of knockout strategy by CRISPR/Cas9. Red: guide RNA (gRNA) targeting sites. **E** Wild-type (WT), heterozygous, and homozygous mutations in CFAP57 were confirmed by PCR (Left panel); and sanger sequencing (Right panel) showing the homozygous mutations, The locations of mutation site is depicted by red box. **F** Expression of CFAP57 protein in WT and *Cfap57*^*M/M*^ testis with GAPDH as a loading control, *n* = 3. **G** Body weight changes in WT and *Cfap57*^*M/M*^ mice, *n* = 6. Average testes weight **H** and testes index **I** of WT and *Cfap57*^*M/M*^ mice, *n* = 6. **J** Average pups of WT and *Cfap57*^*M/M*^ male mice mated with wildtype female mice. *n* = 6. Data were presented as the mean ± SEM. **p < 0.01. **K** Hematoxylin and eosin staining of testis, cauda, and caput epididymis sampled from WT and *Cfap57*^*M/M*^ male mice. Scale bars: 20 μm and 50 μm. **L** Ultrastructure of WT and *Cfap57*^*M/M*^ testes as evidenced by transmission electron microscopy (TEM). Severe axonemal disorganization and aberrant mitochondrial aggregates (red arrowheads). Abbreviations: N, Nuclear; AG, acrosomal granule; Mito, mitochondria; Ac, acrosome; Ax, axoneme. Scale bars: 0.5 μm, 1 μm, and 2 μm

Analysis of RNA profiling data from the Expression Atlas database (https://www.ebi.ac.uk/gxa) revealed that *CFAP57* mRNA transcripts show highest expression levels in human and mouse testis (Supplementary Fig. 2A and 2B), with significant expression also detected in ciliated tissues including fallopian tube, bronchus, lung, and nasopharynx, consistent with its role in both reproductive and respiratory ciliary function [11, 32, 39, 40]. To elucidate the expression profile of CFAP57 across various murine tissues, western blotting was performed. Western blotting analysis using anti-CFAP57 antibody (HPA028623, epitope aa 1165–1250) revealed robust protein expression in mouse testis, with undetectable signals in other examined tissues including brain, heart, lung, kidney, intestine, and ovary (Fig. 3A). The marked difference in signal intensity between testis and other tissues suggests either tissue-specific expression patterns or differential detection sensitivity for potential protein isoforms. We further demonstrated that *Cfap57* mRNA expression in mouse testis commences around postnatal day 14 (P14), rapidly increasing by P21 (Fig. 3B), a timeframe coinciding with the initial appearance of round spermatids. Lower levels of CFAP57 protein were detected from embryonic day 16.5 (E16.5) through P21. The CFAP57 protein level peaked at postnatal day 28 (P28) thereafter maintained a high level (Fig. 3C). Collectively, these findings suggest a functional role for CFAP57 during the later stages of spermatogenesis, and further point to identified mutations in CFAP57 as candidate pathogenic variant in affected individuals.

Given the high sequence homology between murine and human CFAP57, along with the robust expression of CFAP57 in the murine testis, we engineered mice with a point mutation, informed by the first patient’s nonsense mutation in the *CFAP57* gene (*Cfap57*^*R1083X*^, or *Cfap57*^*M/M*^) using CRISPR-Cas9 technology (Fig. 3D). As anticipated, we successfully obtained *Cfap57*^*M/M*^ mice, which were confirmed by genotyping and sanger sequencing (Fig. 3E), and followed by western blotting (Fig. 3F). Body weight, testicular weight, and testicular index analysis did not reveal significant differences between the WT and *Cfap57*^*M/M*^ groups (Fig. 3G and I). Although vaginal plugs were routinely observed in females, no pups were obtained when adult *Cfap57*^*M/M*^ male mice mated with WT female mice for about 3 months (Fig. 3J). H&E staining of testis and epididymis sections showed a striking reduction of spermatids and spermatozoa in the testis and cauda epididymides (Fig. 3K). These results suggest that *Cfap57*^*M/M*^ male mice are infertile.

We further performed transmission electron microscopy (TEM) analysis on testicular sections from both WT and *Cfap57*^*M/M*^ mice. In comparison to WT group, no discernible differences were observed in the nuclear and acrosomal structures of spermatids during the Golgi and Cap phases of spermiogenesis. However, in the *Cfap57*^*M/M*^ mice, spermatid nuclei frequently exhibited abnormal morphology, and axonemal disorganization with loss of the canonical ‘9 + 2’ arrangement, frequent absence the central pair, and the clusters of haphazardly aggregated mitochondria at the maturation phases (Fig. 3L). These findings indicate that the loss of CFAP57 predisposes spermatids to defects during the later differentiation stages of spermiogenesis, particularly affecting nuclear shaping and flagellar assembly.

### *Cfap57*^*M/M*^ sperm exhibit the MMAF phenotype

**Fig. 4:**
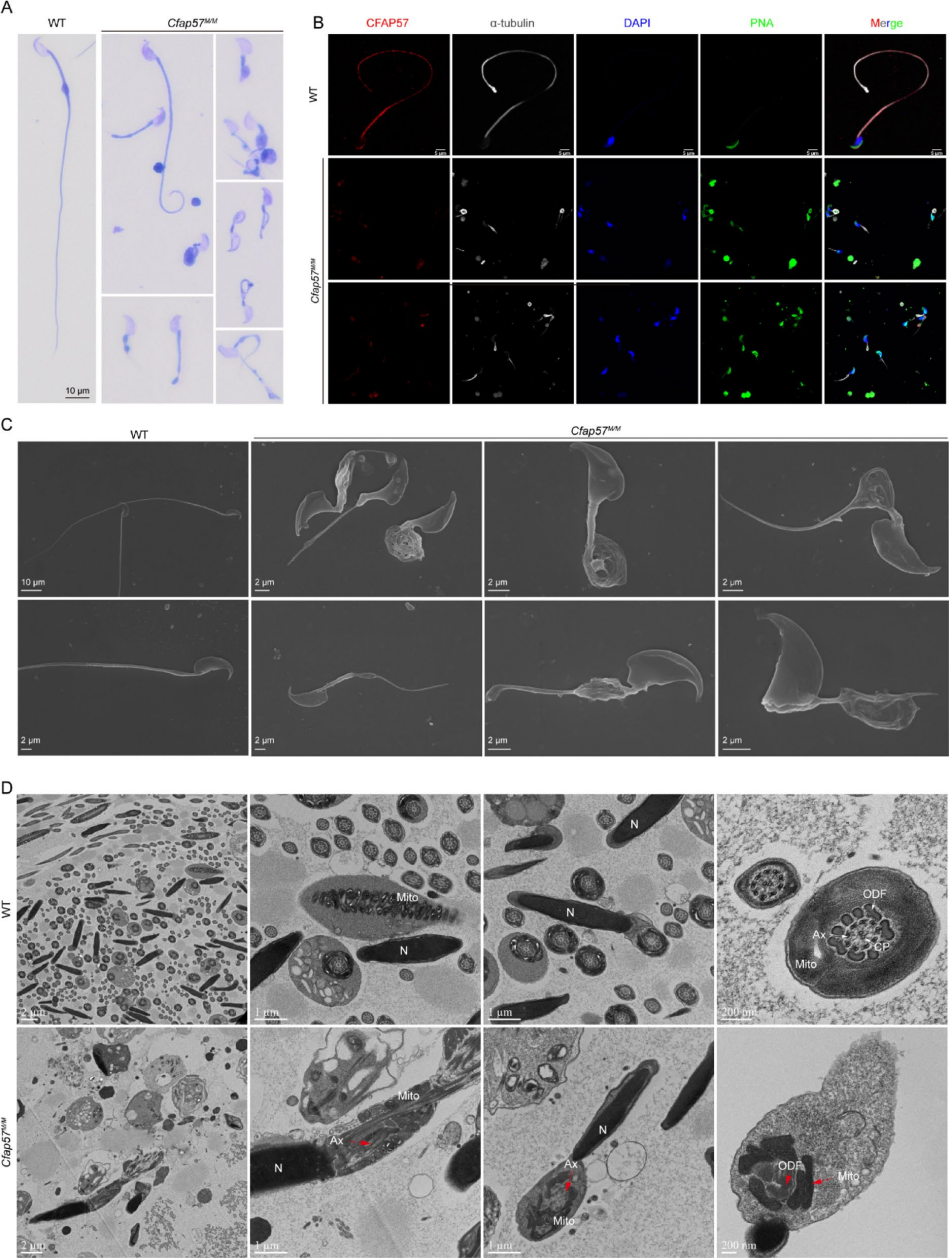
*Cfap57*^*M/M*^ mice exhibit typical MMAF phenotype. **A** Hematoxylin and eosin staining of sperm from WT and *Cfap57*^*M/M*^ male mice. *n* = 3, Scale bars: 10 μm. **B** Immunofluorescence staining of CFAP57 in sperm from WT and *Cfap57*^*M/M*^ mice. CFAP57 (red), α-Tubulin (gray) antibodies and peanut agglutinin (PNA, green) were used. The nuclei of sperm were DAPI labeled (blue). Scale bars: 5 μm. **C** Ultrastructure of spermatozoa determined by SEM from WT and *Cfap57*^*M/M*^ male mice. *Cfap57*^*M/M*^ sperm exhibited severe flagellar malformations. Scale bars: 2 μm and 10 μm. **D** Ultrastructure of sperm in the epididymis determined by TEM from WT and *Cfap57*^*M/M*^ male mice. The axoneme is disorganized and mitochondrial exhibited both aberrant localization patterns and defective assembly processes. The red arrows pointed to these defects. Abbreviations: N, Nuclear; Mito, mitochondria; Ax, axoneme; ODF, outer dense fibers; CP, central pair. Scale bars: 2 μm, 1 μm and 200 nm

To further characterize the spermatogenic defects in *Cfap57*^*M/M*^ mice, we assessed sperm motility, morphology, and fertilizing capacity. Spermatozoa from *Cfap57*^*M/M*^ mice failed to swim out from the cauda epididymis when incubated in HTF medium, in stark contrast to WT controls. These mutant sperm displayed profound immotility and could only be recovered by mincing the epididymal tissue. Consequently, they were incapable of fertilizing oocytes in vitro (data not shown). Phase-contrast microscopy of cauda epididymal spermatozoa revealed that *Cfap57*^*M/M*^ sperm displayed severe flagellar abnormalities, including absence, shortening, coiling, bending, and irregular configurations, as confirmed by hematoxylin and eosin (H&E) staining (Fig. 4A), which indicates that CFAP57 is involved at the stage of sperm flagellar formation. Furthermore, immunofluorescence staining demonstrated that in WT spermatozoa, CFAP57 predominantly localized along the entire length of the flagellum, co-localizing with the axonemal marker α-tubulin. In stark contrast, this CFAP57 signal was markedly reduced or virtually absent in spermatozoa from *Cfap57*^*M/M*^ mice (Fig. 4B). Collectively, the severe morphological flagellar abnormalities in *Cfap57*^*M/M*^ sperm closely recapitulate the MMAF phenotype observed in individuals with biallelic CFAP57 mutations.

Scanning Electron Microscope (SEM) was performed to determine the ultrastructure of aberrant sperm morphology. As compared with WT group, *Cfap57*^*M/M*^ sperm displayed severe malformation of the flagella, most possessed an abnormal flagellum in midpiece (Fig. 4C). Ultrastructural analysis by TEM revealed that while WT spermatozoa displayed normal morphology with well-organized flagellar components including the axoneme, outer dense fibers, and mitochondrial sheath. *Cfap57*^*M/M*^ spermatozoa showed widespread axonemal disorganization—loss of the canonical “9 + 2” pattern, doublet misalignment, central pair defects, and disruption of outer dense fibers and the fibrous sheath. The mitochondrial sheath was also mislocalized and improperly assembled, exhibiting irregular rings and asymmetric distribution (Fig. 4D). Altogether, these results indicate that deletion of *Cfap57* impairs spermatogenesis, resulting in male infertility.

### CFAP57 is associated with MYH10 during flagellogenesis

**Fig. 5:**
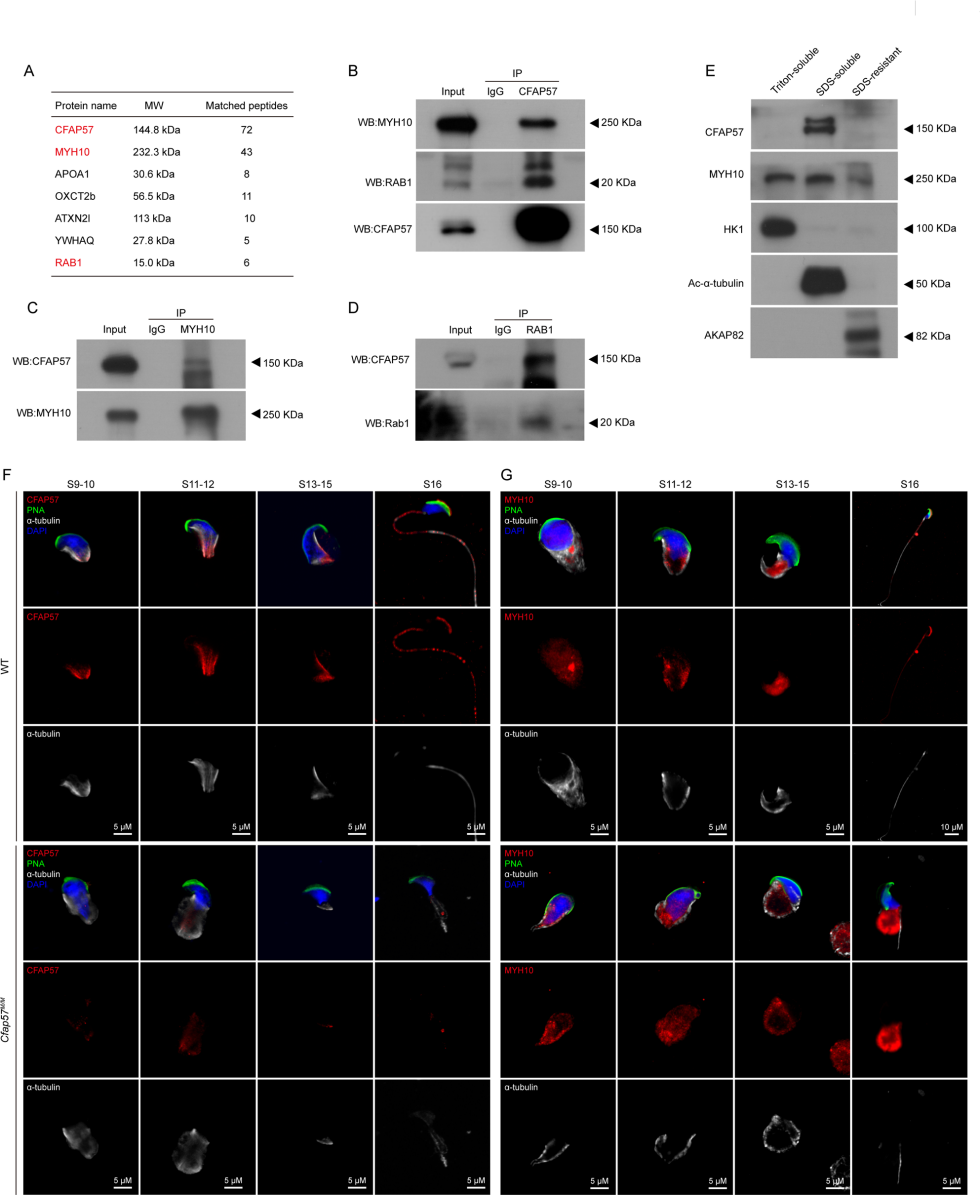
CFAP57 interacts with MYH10 and RAB1. **A** A list of CFAP57-interacting partners in murine testes identified by IP-MS. Proteins were extracted from testis of WT or *Cfap57*^*M/M*^ mice, followed by immunoprecipitation using anti-IgG and CFAP57 antibodies and identified by MS analysis. **B** Co-IP followed by western blotting analysis were performed using lysates extracted from WT testis. Immunoprecipitated proteins by anti-IgG and CFAP57 antibodies were analyzed by western blotting using anti-MYH10, RAB1, and CFAP57 antibody. **C** Immunoprecipitated proteins by anti-IgG and MYH10 antibodies were analyzed by western blotting using anti-MYH10 and CFAP57 antibody. **D** Immunoprecipitated proteins by anti-IgG and RAB1 antibodies were analyzed by western blotting using anti-RAB1 and CFAP57 antibody. All data shown are representative of three independent experiments. **E** Western blotting analysis of fractionated mouse spermatozoa: Triton X-100 soluble, SDS-soluble, and SDS-resistant insoluble fractions. CFAP57 and MYH10 were found in the SDS-soluble fraction. HK1S, acetylated Tubulin, and AKAP82 were used as makers for Triton-soluble, SDS-soluble, and SDS-resistant fractions, respectively. **F** Immunofluorescence staining of CFAP57 in isolated 9–16 stage spermatids from WT and *Cfap57*^*M/M*^ mice. CFAP57 (red), α-Tubulin (gray) antibodies and peanut agglutinin (PNA, green) were used. The nuclei of sperm were DAPI labeled (blue), *n* = 3, Scale bars: 5 μm. **G** Immunofluorescence staining of CFAP57 in isolated 9–16 stage Spermatids from WT and *Cfap57*^*M/M*^ mice. Myh10 (red), α-Tubulin (gray) antibodies and peanut agglutinin (PNA, green) were used. The nuclei of sperm were DAPI labeled (blue), *n* = 3, Scale bars: 5 μm

To elucidate the molecular mechanism underlying CFAP57-mediated regulation of spermiogenesis, we conducted immunoprecipitation-coupled mass spectrometry (IP-MS) using a CFAP57-specific antibody in WT and *Cfap57*^*M/M*^ testicular tissues. Notably, seven potential interacting proteins were exclusively identified in WT testis (Fig. 5A). Subsequently, MYH10 and RAB1 were independently validated through co-immunoprecipitation (Co-IP) experiments. Western bloting analysis revealed that in CFAP57 antibody immunoprecipitates, distinct bands corresponding to CFAP57, MYH10, and RAB1 were consistently detected (Fig. 5B). Reciprocal immunoprecipitation using MYH10 or RAB1 antibodies further confirmed the interactions, demonstrating the presence of CFAP57 and MYH10 bands (Fig. 5C), and CFAP57 and RAB1 bands (Fig. 5D), respectively.

MYH10, a non-muscle myosin II isoform, represents a critical regulatory protein that plays a fundamental role in modulating ciliogenesis [41, 42]. Thus, we consider MYH10 to be a significant target of CFAP57 in the regulation of sperm flagellum formation. Immunofluorescence staining revealed CFAP57 as a flagellar protein (Fig. 4B). To comprehensively characterize the localization of CFAP57 and MYH10, we performed a systematic protein fractionation of sperm cellular components. It showed that CFAP57 was exclusively detected in the SDS-soluble fraction, which was consistent with the axonemal marker α-tubulin (Fig. 5E). MYH10 was widely present in all three fractions (Fig. 5E). These findings strongly suggest that MYH10 is localized within the sperm axoneme.

Next, to further analyze CFAP57 and MYH10 localization during spermatogenesis, we isolated spermatids from the testis and performed IF staining, which demonstrated that CFAP57 mainly localized in the cytoskeleton with α-tubulin during step 1 to step 6, and began polarized localization in the manchette and flagellum from step 9 to step 16 in WT group spermatids; in contrast, CFAP57 was decreased or disappeared dramatically in *Cfap57*^*M/M*^ group spermatids (Fig. 5F and S3A). MYH10 mainly localized in the nuclear from step 1 to step 6 in WT group, which was comparable with *Cfap57*^*M/M*^ group spermatids (Figure S3B); but MYH10 translocated to the manchette and flagellum from step 9 to step 16 in WT group spermatids, and MYH10 often mislocated along the flagellum from step 13 in *Cfap57*^*M/M*^ group spermatids (Fig. 5G). These results suggest that CFAP57 is associated with MYH10 during flagellogenesis.

### MYH10 localized in flagella

**Fig. 6:**
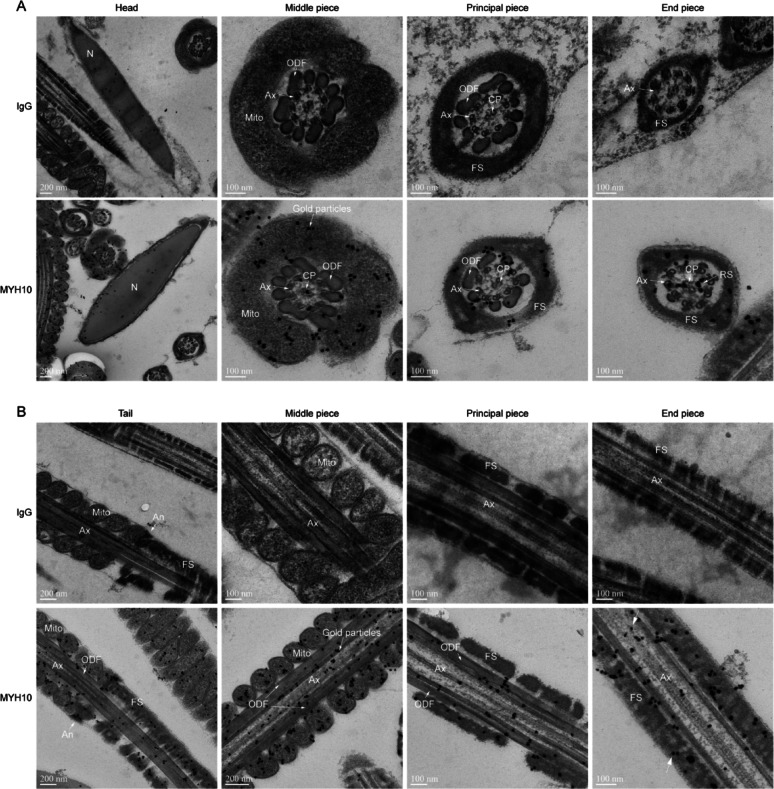
MYH10 is a pan-axonemal localization protein in sperm flagellum. **A** IEM images of IgG and MYH10 the cross-section of mouse sperm. Arrows indicate MYH10 gold particles. **B** IEM images of IgG and MYH10 the longitudinal section of mouse sperm. Red arrows indicate MYH10 gold particles. N, Nuclear; Mito, mitochondria; Ax, axoneme; ODF, outer dense fibers; CP, central pair; RS, radial spokes; FS, Fibrous sheath. Scale bars: 100 nm, and 200 nm

To further make sure the subcellular localization of MYH10, we performed immunoelectron microscopy (IEM) in sperm, which clearly showed that gold particles conjugated to the MYH10 antibody were predominantly detected in mitochondrial sheaths and flagellar ultrastructure, including the axonemal (Ax), central pair (CP), outer dense fibers (ODF), radial spokes (RS) and fibrous sheath (FS) in the cross-section (Fig. 6A) and longitudinal section (Fig. 6B) in sperm. These results demonstrate that MYH10 is a pan-axonemal localization protein in sperm flagellum.

### Defect in CFAP57 induce MYH10 mis-localization in mouse and human sperm

**Fig. 7:**
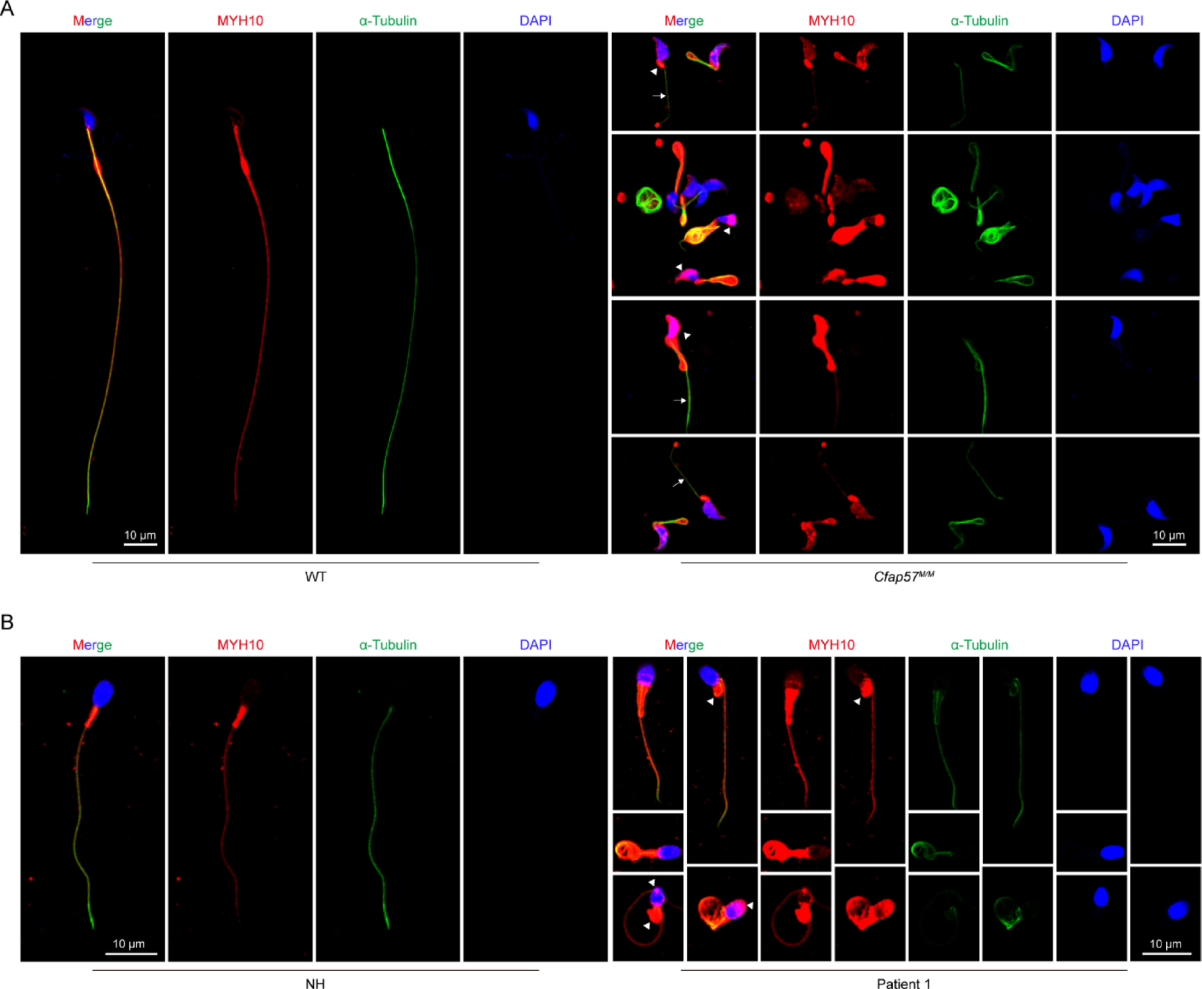
Defect in CFAP57 induce MYH10 mis-localization in mouse and human sperm. **A** Immunofluorescence staining of MYH10 in sperm from WT and *Cfap57*^*M/M*^ mice. MYH10 (red), α-Tubulin (green) antibodies were used. The nuclei of sperm were DAPI labeled (blue), *n* = 3, Scale bars: 10 μm. **B** Immunofluorescence staining of MYH10 in sperm from NH and P1 with CFAP57 mutations. MYH10 (red), α-Tubulin (green) were used. The nuclei of sperm were DAPI labeled (blue), *n* = 3, Scale bars: 10 μm

Immunofluorescence staining showed that MYH10 indeed expressed in mitochondrial sheaths and flagellum in WT cauda epididymis sperm, which was colocalized with α-tubulin. But the mislocalized MYH10 protein aggregates in the sperm head, mitochondrial sheath, with missing regions in the flagellar portion in *Cfap57*^*M/M*^ group sperm (Fig. 7A). We further found that MYH10 also mislocalized in the sperm head and mitochondrial sheath in human CFAP57 mutation individual (Fig. 7B). Pronounced MYH10 mislocalization in *Cfap57*^*M/M*^ mouse sperm compared to human CFAP57-mutant sperm suggests species-specific differences in genetic background and compensatory mechanisms. This observation is consistent with more severe flagellar phenotypes reported in mouse CFAP57 models versus human patients [11]. These results suggest that CFAP57 may like a scaffold protein to make sure the right position of MYH10 during flagellogenesis.

### Defect in CFAP57 disrupt IFT88 induced flagellar assembly and maintenance in mouse and human sperm

**Fig. 8:**
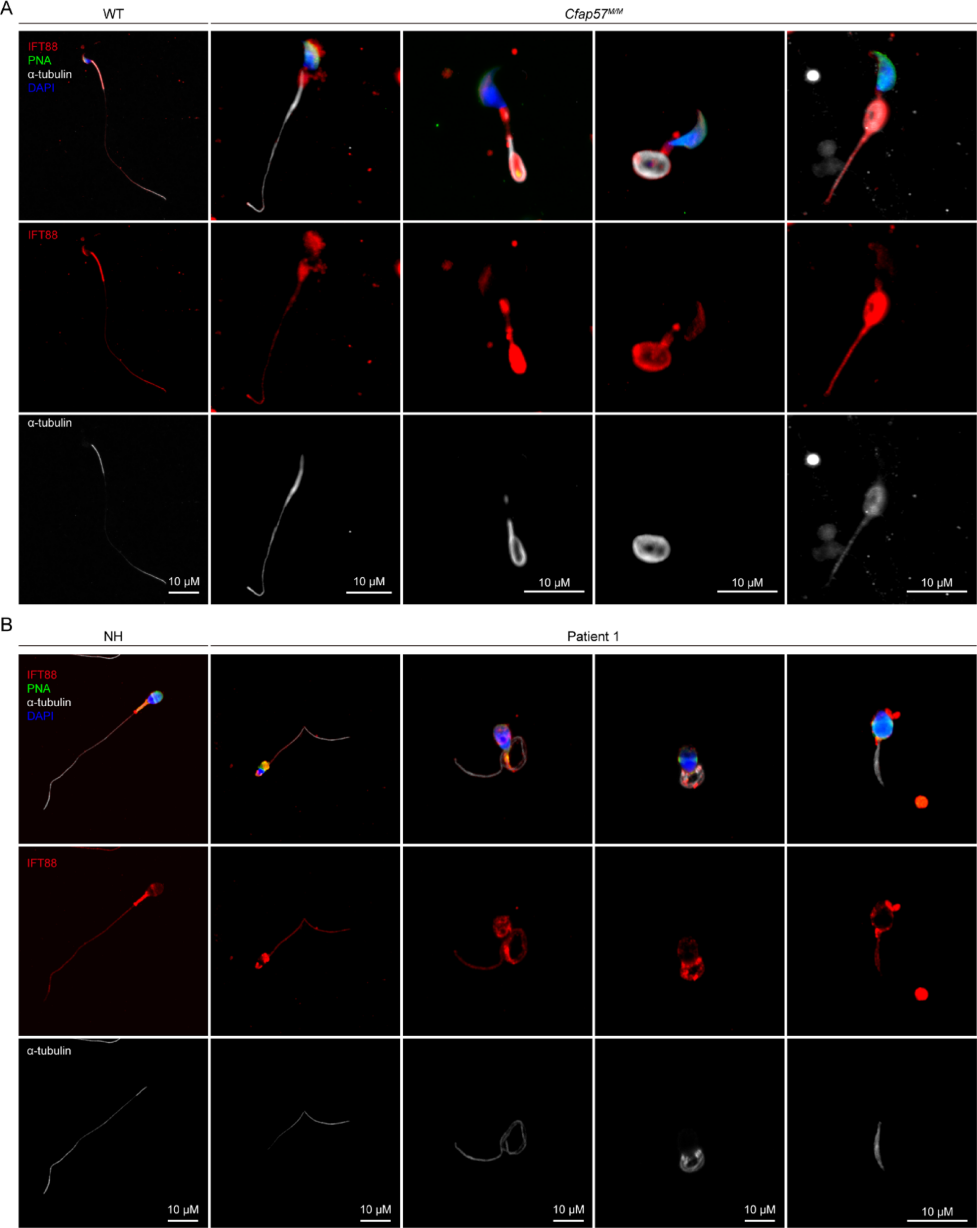
A defect in CFAP57 disrupts the localization of IFT88 in both mouse and human sperm. **A** Immunofluorescence staining of IFT88 in sperm from WT and *Cfap57*^*M/M*^ mice. IFT88 (red), α-Tubulin (gray) antibodies were used. The nuclei of sperm were DAPI labeled (blue), *n* = 3, Scale bars: 10 μm. **B** Immunofluorescence staining of IFT88 in sperm from NH and P1 with CFAP57 mutations. IFT88 (red), α-Tubulin (gray) antibodies were used. The nuclei of sperm were DAPI labeled (blue), *n* = 3, Scale bars: 10 μm

Intraflagellar transport (IFT) is fundamental to the biogenesis of cilia and flagella in eukaryotic cells. IFT is performed by the IFT-A and IFT-B protein complexes, which together link cargoes to the microtubule motors kinesin and dynein [43]. IFTs proteins play fundamental roles during spermatogenic, facilitating flagellar assembly and cellular remodeling. IFT88 is a crucial component of the IFT-B subcomplex, male Ift88^−/−^ mice is infertile [44]. Importantly, MYH10 facilitates centrosomal recruitment of IFT88, which is required for transporting structural components to the ciliary tip [45]. Therefore, we detect the expression of IFT88 in isolated spermatids by IF, the results showed that IFT88 was colocalized with α-tubulin in cytoplasmic from step 1 to step 8, whether in the WT or *Cfap57*^*M/M*^ group; In later stage of spermatogenesis, IFT88 proteins begin to accumulate unilaterally with the onset of sperm elongation, gradually expressing in the manchette and flagellum, and subsequently localizing in the mitochondrial sheath and flagellum during sperm maturation in WT group. In contrast, in the *Cfap57*^*M/M*^ group, IFT88 proteins exhibit an aberrant phenotypic distribution, with abnormal aggregation and mislocalization in the manchette, mitochondrial sheath and flagellum (Figure S4). In cauda epididymis sperm, in addition to localization in the flagellum and mitochondrial sheath, IFT88 is also expressed in the acrosome of the sperm head in WT group. But it was abnormal aggregation in mitochondrial sheath, and mislocalization in the sperm head and flagellum in the *Cfap57*^*M/M*^ group (Fig. 8A). These results suggest that CFAP57 could be involved in the proper localization of MYH10 and IFT88. We further detected the expression of IFT88 protein in patient with CFAP57 mutation sperm, which showed the same defect with mice, including IFT88 mislocalization in the sperm head, mitochondrial sheath and flagellum (Fig. 8B).

Altogether, we confirm that CFAP57 plays a crucial role in sperm flagellum formation. Mechanistically, CFAP57 primarily mediates its function through protein interactions with MYH10, enabling correct localization at key sites during spermatogenesis, including the manchette, mitochondrial sheath, and flagellum. Given MYH10’s role in centrosomal recruitment of IFT88 [45], it is plausible that MYH10 recruits IFT88 to accumulate at these sites, thereby orchestrating sperm flagellum formation. Protein functional mutations in CFAP57 lead to abnormal localization of MYH10 and IFT88, consequently causing flagellum formation defects in both humans and mice, resulting in MMAF.

### ICSI can overcome the infertility caused by CFAP57 mutation

While previous studies have established that CFAP57 mutations impair male fertility, effective clinical treatments remain unavailable. For Patient 1 in this study, assisted fertilization was performed using intracytoplasmic sperm injection (ICSI). Following an antagonist protocol, his wife had 14 oocytes retrieved. Twelve oocytes were injected, resulting in 11 viable day-2 embryos, with 5 blastocysts formed (Table 2). After frozen-thawed embryo transfer (FET) of one high-quality blastocyst, a clinical pregnancy was achieved (Table 2). These results suggest that patients with MMAF resulting from *CFAP57* variants can be effectively treated with ICSI.

**Table 2:** Outcomes of ICSI treatment in patient 1

Patient 1	
Male age (years)	39
Female age (years)	36
Cycles (n)	1
Oocytes collected (n)	14
Metaphase II oocytes (n)	13
Maturation oocyte rate (%)	12
2 PN (n)	11
Fertilization rate (%)	78.57
Blastocysts (n)	5
Num. of transferred embryos (n)	1
Clinical pregnancy (n)	1
Implantation rate (%)	100
Delivery (n)	1

## Discussion

Previous reports have confirmed that cilia and flagella-associated protein (CFAP) family members play pivotal roles in sperm flagellum formation, a process crucial for sperm motility and male fertility. Notably, defects in these genes contribute to MMAF in both mice and human patients [9–17]. CFAP57 belongs to the CFAP family and was highly conserved across mammalian species [32]. Recently, Ma et al. identified two homozygous stop-gain mutations in *CFAP57* (c.2872 C >T, c.2737 C >T) in 3 Pakistani families; They found that loss-of-function mutations in CFAP57 selectively abrogate the long isoform in sperm, disrupting inner dynein arm (particularly single-headed IDA) docking/assembly without broadly reducing dynein protein abundance, thereby impairing axonemal waveform generation and motility to produce the MMAF phenotype [11]. Here, we found three novel mutations [c.3250 C >T (p.R1084X), c.1340T >C (p.V447A), c.1856G >A (p.R619H)] in CFAP57 from male infertility in Chinese patients. Consistent with the previous result, we found that these mutations of CFAP57 also resulted in MMAF. Additionally, we demonstrated that the P1 site corresponds to the long transcript, while the P2 site corresponds to both the long and short transcripts. However, both lead to MMAF and do not cause PCD. Ultrastructural analysis shows that the P1 mutation results in disorganization of the axonemal structure, with disruption of the “9 + 2” microtubule arrangement and disordered mitochondrial distribution. Most importantly, Co-immunoprecipitation experiments indicate that CFAP57 is associated with MYH10, and MYH10 may recruit IFT88 to orchestrate sperm flagellum formation.

CFAP57 has four transcript isoforms according to NCBI: NM_001195831.3 (1-1250aa), NM_001378189.1 (1-1250aa), NM_001167965.1 (1-1239aa), and NM_152498.3 (1-698aa). Based on Ma et al. [11], the long transcript (NM_001195831.3) is essential for sperm flagellar assembly, while the short transcript (NM_152498.3) is critical for respiratory ciliary function [32]. In this study, clinical evaluation revealed no evidence of primary ciliary dyskinesia (PCD) in either patient. The P1 mutation, located near the site reported by Ma et al. [11], produces similar human and mouse phenotypes with abnormal sperm morphology. Although the P2 mutation site is adjacent to that described by Bustamante-Marin et al. [32], the mutation types differ—compound heterozygous versus truncating mutations—resulting in distinct CFAP57 functional effects. Notably, P2 mutations cause spermatogenesis defects without PCD, suggesting selective impairment of the long transcript isoform. These findings indicate that despite variable effects on different CFAP57 isoforms, both mutations converge on a tissue-specific phenotype affecting sperm flagellar function, resulting in MMAF without respiratory ciliary impairment.

Sperm morphogenesis involves spermatid polarity changes and cytoskeletal reorganization, forming manchette and flagellar structures essential for motility and fertility. The axoneme features a conserved “9 + 2” organization—nine peripheral doublet microtubules surrounding two central singlets. Defects in this microtubule complex cause MMAF. It has been reported that the loss of CFAP57 would cause primary ciliary dyskinesia [32, 39, 46]. However, neither patient in this study presented with PCD. In our results, axonemal structures appeared disorganized in CFAP57 mutation sperm in a significant portion of human and mouse by SEM and TEM analysis. We also noticed that the mitochondrial assembly defects in CFAP57 mutation sperm. These results confirm the essential role of CFAP57 in axonemal structural organization, consistent with previous findings by Ma et al. [11].

Microtubules, formed by tubulin, are the key components of the axoneme in cilia and flagella. Recent cryo-EM identified that CFAP57 was identified as a potential structural component of the DMT A3 and A4, spanning approximately 96 nm along the DMT [46]; CFAP57 connects the N-terminal region to the I1 intermediate/light chain domain and positions the C-terminus near the radial spoke RS1 [46]. Our IF results supported this cryo-electron microscopy results that CFAP57 and α-tubulin exhibit a highly consistent co-localization during spermatogenesis, particularly in elongating spermatids in the later stages of flagellogenesis.

CFAP57 was an inner-arm dynein (IDA) adaptor and contacted multiple IDA subtypes, including CFAP45, CCDC96/113, CFAP337, and dyneins g and d, establishing it as a critical regulatory hub in the assembly of these dyneins [19, 39, 46, 47]. A recently published result indicates that CCDC113 is essential for male fertility as it stabilizes the sperm axoneme and head-tail coupling apparatus by interacting with proteins CFAP57 and CFAP91 [19]. To identify novel proteins interacting with CFAP57, we utilized IP-MS and discovered that MYH10 and RAB1 can interact with CFAP57. This finding has been validated through co-immunoprecipitation (Co-IP) experiments. We notice that MYH10 is a non-muscle myosin II isoform, emerges as a critical regulator of ciliogenesis through its multifaceted roles in cellular dynamics [41, 42, 45, 48]. A prior RNAi library screen identified MYH10 as a positive regulator of ciliogenesis [41]. MYH10 regulates ciliogenesis through its interaction with the microtubule acetyltransferase Mec17, which enhances MYH10 expression and subsequently promotes the formation of a pericentrosomal membranous compartment [42]. MYH10 promotes cortical actin levels and influences centrosomal recruitment of IFT88, thereby facilitating cilium assembly [45]. Genetic ablation of MYH10 significantly affect mitochondrial DNA copy number in mouse embryonic fibroblasts [48]. Intraflagellar transport (IFT) proteins are essential for mammalian spermatogenesis. IFT88, a key component of the IFT-B complex, plays a crucial role in the assembly of mouse sperm flagella, as its absence leads to defects in flagellar structure and male infertility [44, 49] In this study, Our IF and IEM analysis confirmed MYH10 pan-axonemal localization in sperm tail. The IEM results also clearly showed that MYH10 localized in mitochondrial. These results suggested that MYH10 probably plays a more important role in sperm Mitochondrial organization and flagellum formation.

While MYH10 has not been linked to MMAF in humans, recent studies show heterozygous MYH10 variants cause neurodevelopmental disorders and congenital anomalies, indicating its critical role in primary cilia formation [50]. MYH10’s role in flagellogenesis is understudied, but SPATA6-MYH10 interactions facilitate cytoskeletal transport and connecting piece assembly during spermatogenesis [51]. In this study, IF staining of mouse spermatids showed CFAP57, MYH10, and IFT88 colocalization with α-tubulin in the manchette and flagellum (steps 9–16). CFAP57 mutation causes MYH10 and IFT88 mislocalization, leading to axonemal disorganization and reduced fertility. Similarly, MYH10 mislocalization disrupts actin networks in patient cells and causes developmental defects in zebrafish models [52]. Proper MYH10 localization is essential for cellular function, as dysfunction causes defects in sperm flagella and eyes. CFAP57, MYH10, and IFT88 participate in cytoskeletal remodeling, organelle positioning, and flagellar protein trafficking, forming a complex mechanism essential for spermatogenesis.

Although, the mutation of CFAP57 lead the detects in sperm flagellum and motility, the patient obtained healthy offspring through ICSI. To our knowledge, this represents the first published account of ICSI outcomes in patient with CFAP57 mutations. Therefore, our findings strongly support ICSI as the treatment of choice for patients with CFAP-associated MMAF.

While our study provides significant insights into CFAP57’s role in male fertility, certain limitations should be noted. The unavailability of patient samples for the P2 variant limited our ability to perform direct morphological and functional validations. Additionally, our investigation lacked systematic perturbation analyses for each CFAP57 isoform, and the absence of differential rescue experiments targeting individual transcripts represents a notable gap in dissecting the functional contributions of alternative splice variants. Future studies addressing these limitations, particularly the acquisition of additional patient samples and isoform-specific functional analyses, would further strengthen our understanding of CFAP57’s role in male infertility.

## Conclusion

In summary, CFAP57 mutation led to MMAF in human and mice with aberrant sperm flagella. We discovered a new pan-axonemal protein, MYH10, which associated with CFAP57 and may recruit IFT88 to flagella. Our research also demonstrated that CFAP57 functions as an axonemal protein that acts as a suitable adaptor for the proper localization of MYH10 on the axoneme; subsequently, MYH10 may recruit IFT88 to the flagella, promoting flagella assembly. Furthermore, we demonstrated that ICSI is an effective method to rescue CFAP57-associated male infertility. Therefore, our findings provide novel insights into the molecular basis of sperm flagellogenesis and offer a valuable treatment option for CFAP57-associated male infertility.

## Electronic Supplementary Material

Below is the link to the electronic supplementary material.

Supplementary Material 1.

Supplementary Material 2.

Supplementary Material 3.

## Data Availability

No datasets were generated or analysed during the current study.
