# Supplementary material for: Mutations in CFAP57 disrupt the localization of MYH10 and IFT88, leading to flagellogenesis failure in humans and mice
Source: Hum Genomics. 2025 Dec 29;19:152. doi: 10.1186/s40246-025-00859-x (PMC12751231; doi:10.1186/s40246-025-00859-x)
Supplement: Supplementary file 1 — Supplementary Material 1. [file 40246_2025_859_MOESM1_ESM.docx]

**Supplementary Material**

**Article title: Mutations in CFAP57 Disrupt the Localization of MYH10 and IFT88, Leading to Flagellogenesis Failure in Humans and Mice**

**Journal name**: Human Genomics

**Author name:** Yongjie Chen^1, #*^, Lin Li^1, *^, Ranran Meng^2^, Shanze Li^2^, Yuhua Li^2^, Zhaodi Jiang^2^, Dan Xu^2^, Zhao Lu^3^, Chenghong Yin^1#^, Yanwei Sha^4,5,6#^, Fengchao Wang^2#^

**Author afiliation:**

1 Central Laboratory, Beijing Obstetrics and Gynecology Hospital, Capital Medical University. Beijing Maternal and Child Health Care Hospital, Beijing, 100026, China.

2 National Institute of Biological Sciences, Beijing, 102206, China. Tsinghua Institute of Multidisciplinary Biomedical Research, Tsinghua University, Beijing, 102206, China

3 Capital Medical University, Beijing, China.

4 Department of Reproductive Medicine, Department of Obstetrics and Gynecology, Women and Children’s Hospital, School of Medicine, Xiamen University, Xiamen, China.

5 Xiamen Key Laboratory of Reproduction and Genetics, Xiamen, China.

6 Fujian Provincial Key Laboratory of Reproductive Health Research, Xiamen University, Xiamen, Fujian, China.

^*^ These authors contributed equally to this work.

**#** **Corresponding author’s email:**

Yongjie Chen (chenyongjie@ccmu.edu.cn), Chenghong Yin (yinchh@ccmu.edu.cn), Yanwei Sha (shayanwei928@126.com) or Fengchao Wang (wangfengchao@nibs.ac.cn)


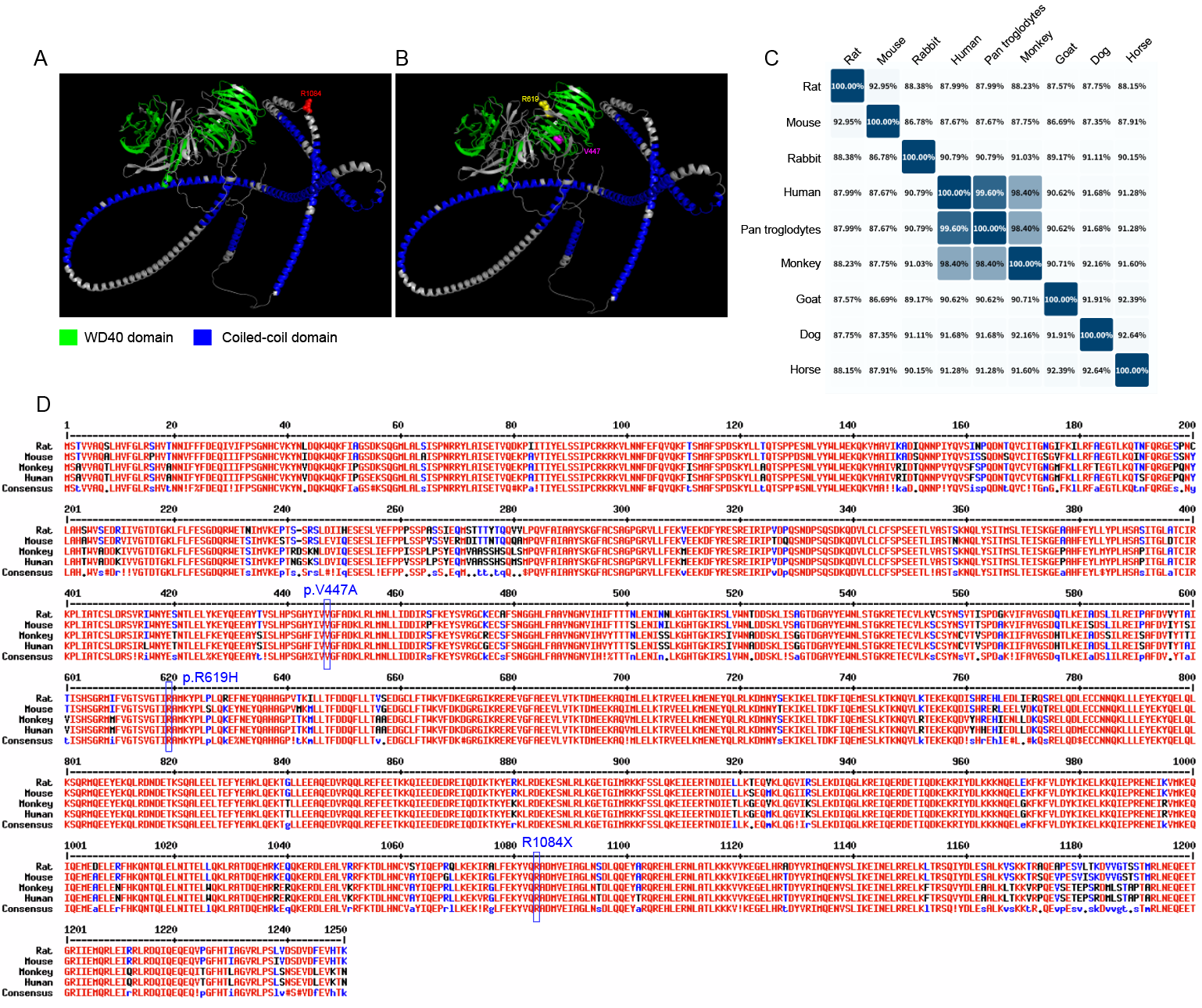


**Fig. S1** **CFAP57 protein domain architecture, mutation mapping, and cross-species conservation analysis.** (A) AlphaFold2 predicted structure of human CFAP57 with mutation sites highlighted. R1084 (red) in inter coiled-coil domain region; (B) V447 (magenta) was located within the WD40 domain, whereas R619 (yellow) was positioned outside the WD40 domain. Predicted structural domains are color-coded: WD40 repeats (green) and coiled-coil domains (blue). (C) Analysis of the similarity of CFAP57 protein sequences across different species (https://www.uniprot.org/). (D) MultAlin sequence alignment showing the differences and similarities of CFAP57 in different species (http://multalin.toulouse.inra.fr/multalin/multalin.html).


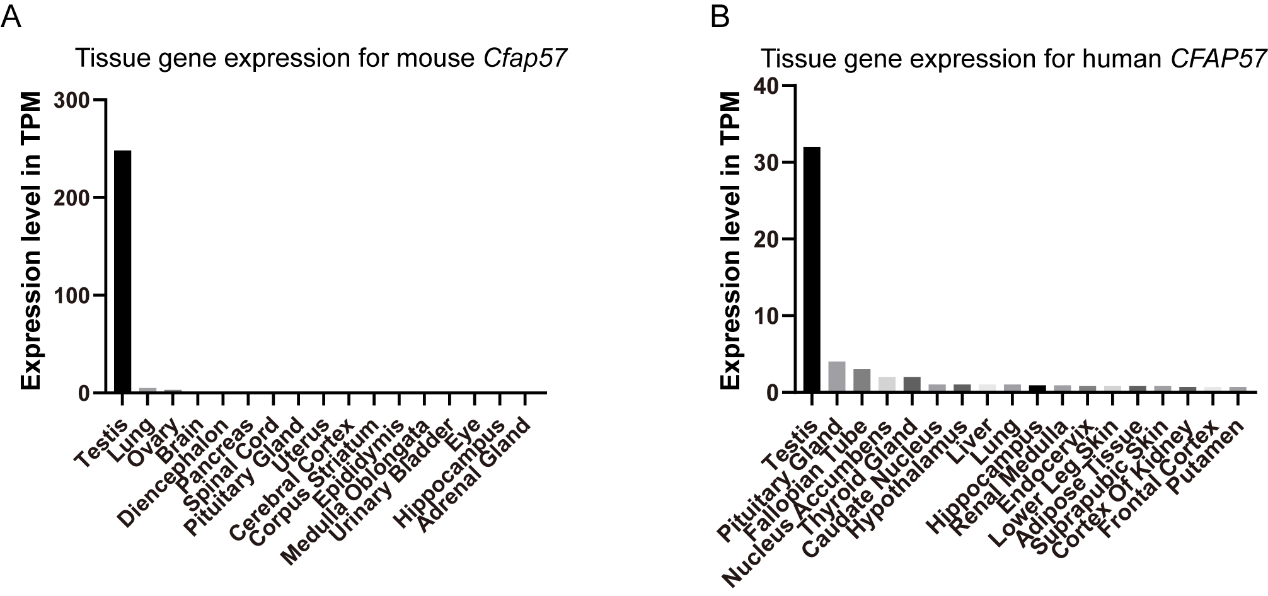


**Fig. S2** ***CFAP57* was highly expressed in testis**. The tissue data for mRNA expression were obtained from the Expression Atlas database (https://www.ebi.ac.uk/gxa). moues (A) and Human (B) *CFAP57* mRNA was specifically expressed in testes.


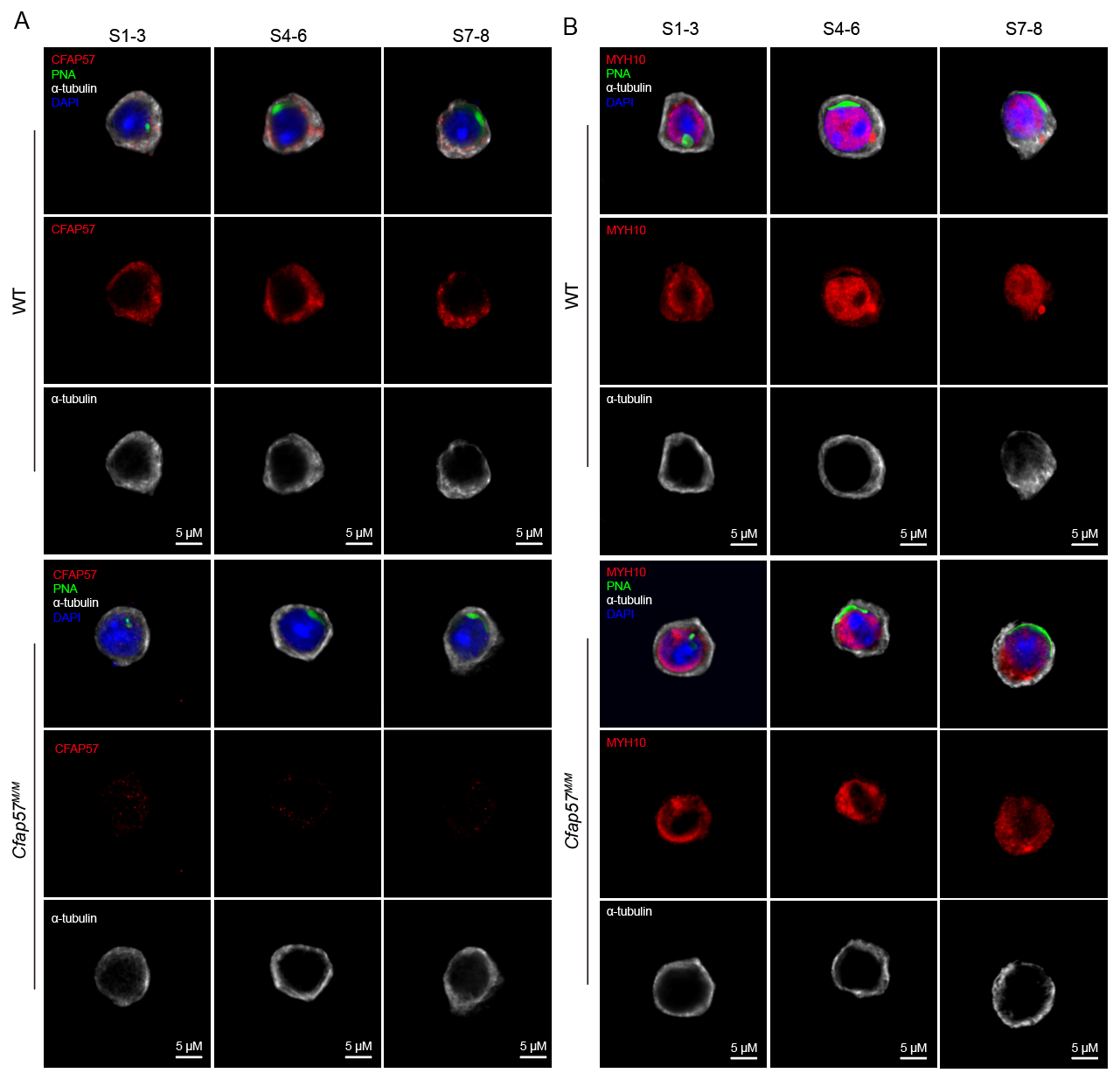


**Fig. S3** **CFAP57 and MYH10 were expressed in isolated spermatids**. (A) Immunofluorescence staining of CFAP57 in isolated spermatids from WT and *Cfap57^M/M^* mice. CFAP57 (red), α-Tubulin (gray) antibodies were used. The nuclei of sperm were DAPI labeled (blue), n=3, Scale bars: 5 μm. (B) Immunofluorescence staining of MYH10 in isolated spermatids from WT and *Cfap57^M/M^* mice. MYH10 (red), α-Tubulin (gray) antibodies were used. The nuclei of sperm were DAPI labeled (blue), n=3, Scale bars: 5 μm.


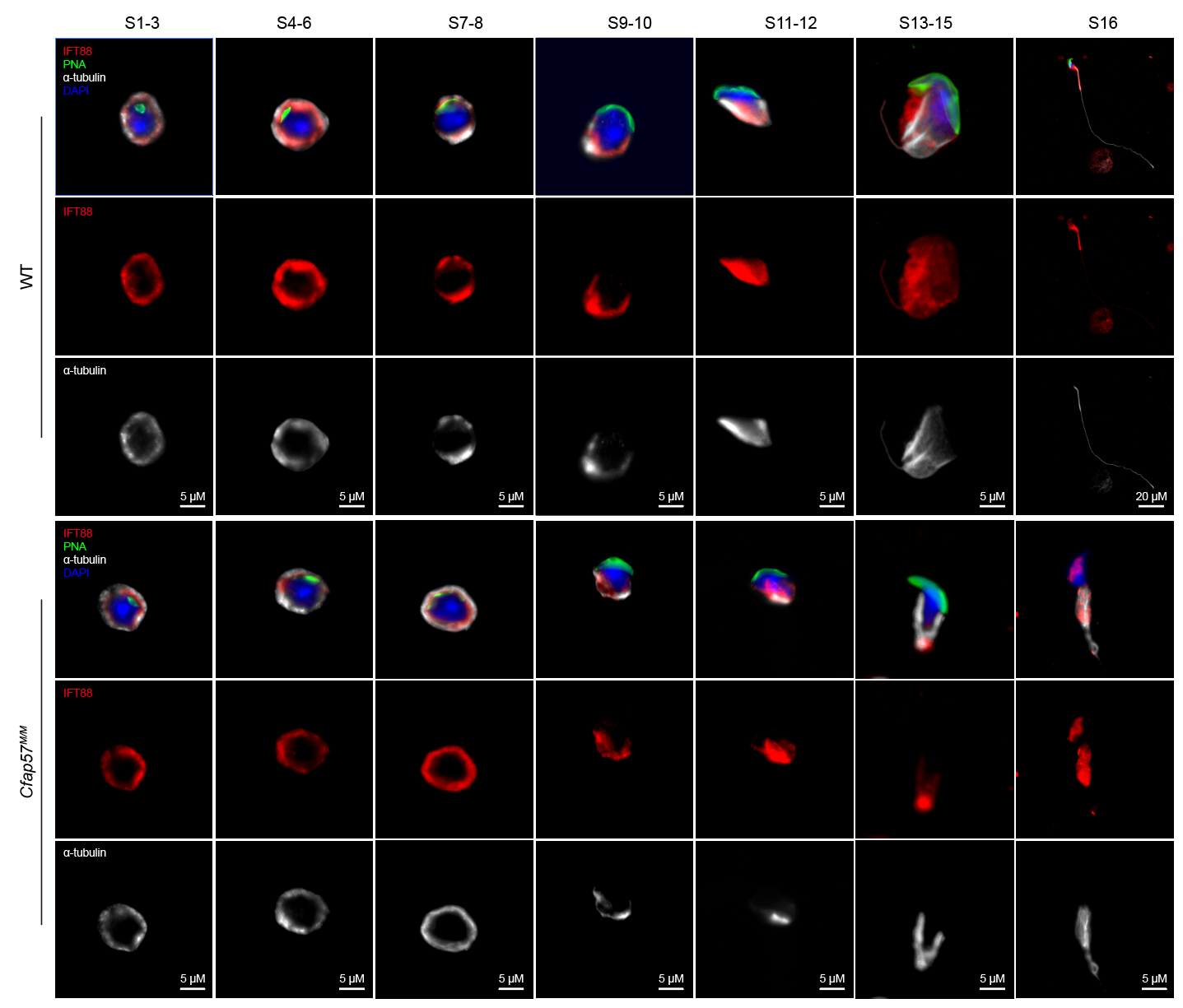


**Fig. S4** **IFT88 was expressed in isolated spermatids**. Immunofluorescence staining of IFT88 in isolated spermatids from WT and *Cfap57^M/M^* mice. IFT88 (red), α-Tubulin (gray) antibodies were used. The nuclei of sperm were DAPI labeled (blue), n=3, Scale bars: 5 μm and 20μm.
