# Supplementary material for: Mutations in CFAP57 disrupt the localization of MYH10 and IFT88, leading to flagellogenesis failure in humans and mice
Source: Hum Genomics. 2025 Dec 29;19:152. doi: 10.1186/s40246-025-00859-x (PMC12751231; doi:10.1186/s40246-025-00859-x)
Supplement: Supplementary file 2 — Supplementary Material 2. [file 40246_2025_859_MOESM2_ESM.docx]

**Table S1. PCR Primers used in Sanger sequencing validation.**

| **Genes and variants** | **Forward Primer** | **Reversed Primer** |
| --- | --- | --- |
| *CFAP57*: c.C3250 >T | TACAGCACCAGAAGGAAACG | GGAATCGGGTATGTGGAGAA |
| *CFAP57*: c.T1340 >C | ATCTTTCTCCCAGGACATCA | AGACAGGACTTATCATAGCG |
| *CFAP57*: c.G1856 >A | GAGGGTTTCGTGAGGATTGA | CATTGGGAGGTTGAAGGGAT |
