# Supplementary material for: Mutations in CFAP57 disrupt the localization of MYH10 and IFT88, leading to flagellogenesis failure in humans and mice
Source: Hum Genomics. 2025 Dec 29;19:152. doi: 10.1186/s40246-025-00859-x (PMC12751231; doi:10.1186/s40246-025-00859-x)
Supplement: Supplementary file 3 — Supplementary Material 3. [file 40246_2025_859_MOESM3_ESM.docx]

**Table S2. Primers were used in mouse genotyping**

| Primer name | 5' to 3' sequence |
| --- | --- |
| CFAP57-mut-screen-F1: | CGCCAGCAGTCAGGTGTTCATCC |
| CFAP57-mut-screen-R1: | CGCTCAGAGCTCACCATGTCTGCTTA |
| CFAP57-WT-R: | TCAGAGCTCACCATGTCTGCCCG |
| CFAP57-mut-screen-F2: | TGGCCTCTTTGAGAAGTACGTGCAGTAA |
| CFAP57-mut-screen-R2: | TGATGCGGACATAGTCGGTGCG |
